# Supplementary material for: Peptide-Targeted Polyplexes for Aerosol-Mediated Gene Delivery to CD49f-Overexpressing Tumor Lesions in Lung
Source: Mol Ther Nucleic Acids. 2019 Oct 18;18:774–86. doi: 10.1016/j.omtn.2019.10.009 (PMC6861568; doi:10.1016/j.omtn.2019.10.009)
Supplement: Document S1. Figures S1–S6 and Supplemental Materials and Methods [file mmc1.pdf]

OMTN, Volume *18*

Supplemental Information

Peptide-Targeted Polyplexes for Aerosol-Mediated Gene Delivery to CD49f-Overexpressing Tumor Lesions in Lung

Alexander Taschauer, Wolfram Polzer, Fatih Alioglu, Magdalena Billerhart, Simon Decker, Theresa Kittelmann, Emanuela Geppl, Salma Elmenofi, Martin Zehl, Ernst Urban, Haider Sami, and Manfred Ogris

Supplemental Figures

Supp. Figure 1

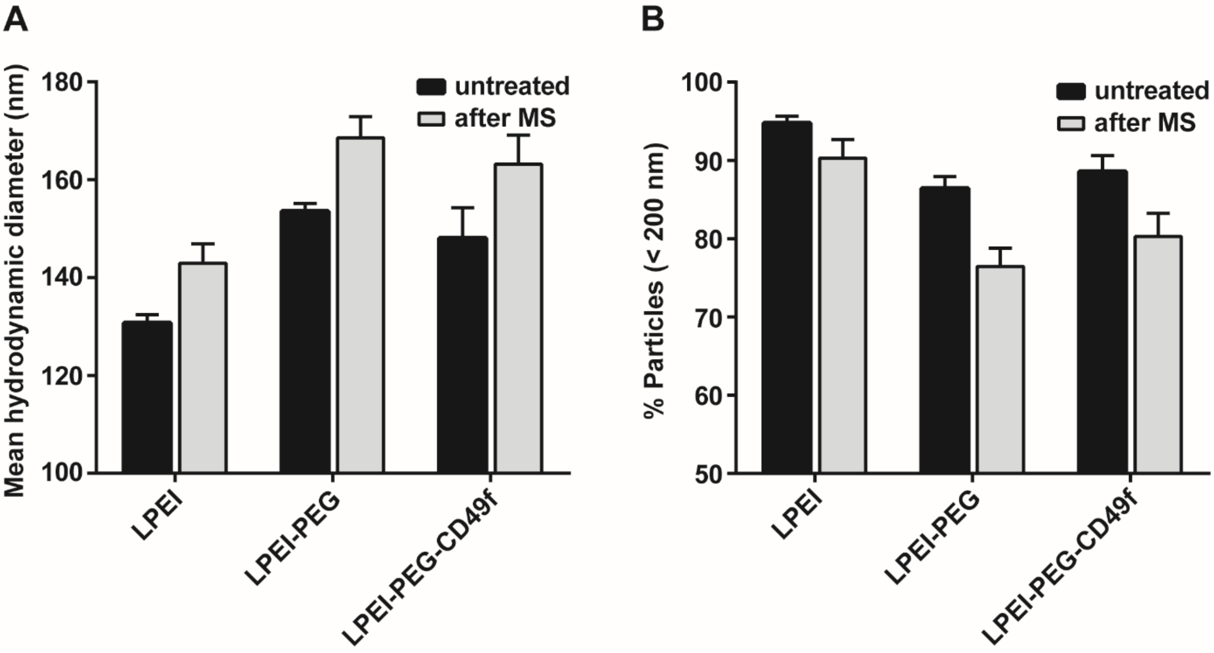

Supp. Figure 1: NTA analysis of polyplexes after aerosolization. Both mean hydrodynamic diameter (A) and percentage of particles <200 nm (B) were evaluated with polyplexes based on LPEI, LPEI-PEG and LPEI-PEG-CD49f at N/P 9 before and after aerosolization. Values are depicted as mean values (n=3 + stddev).

Supp. Figure 2

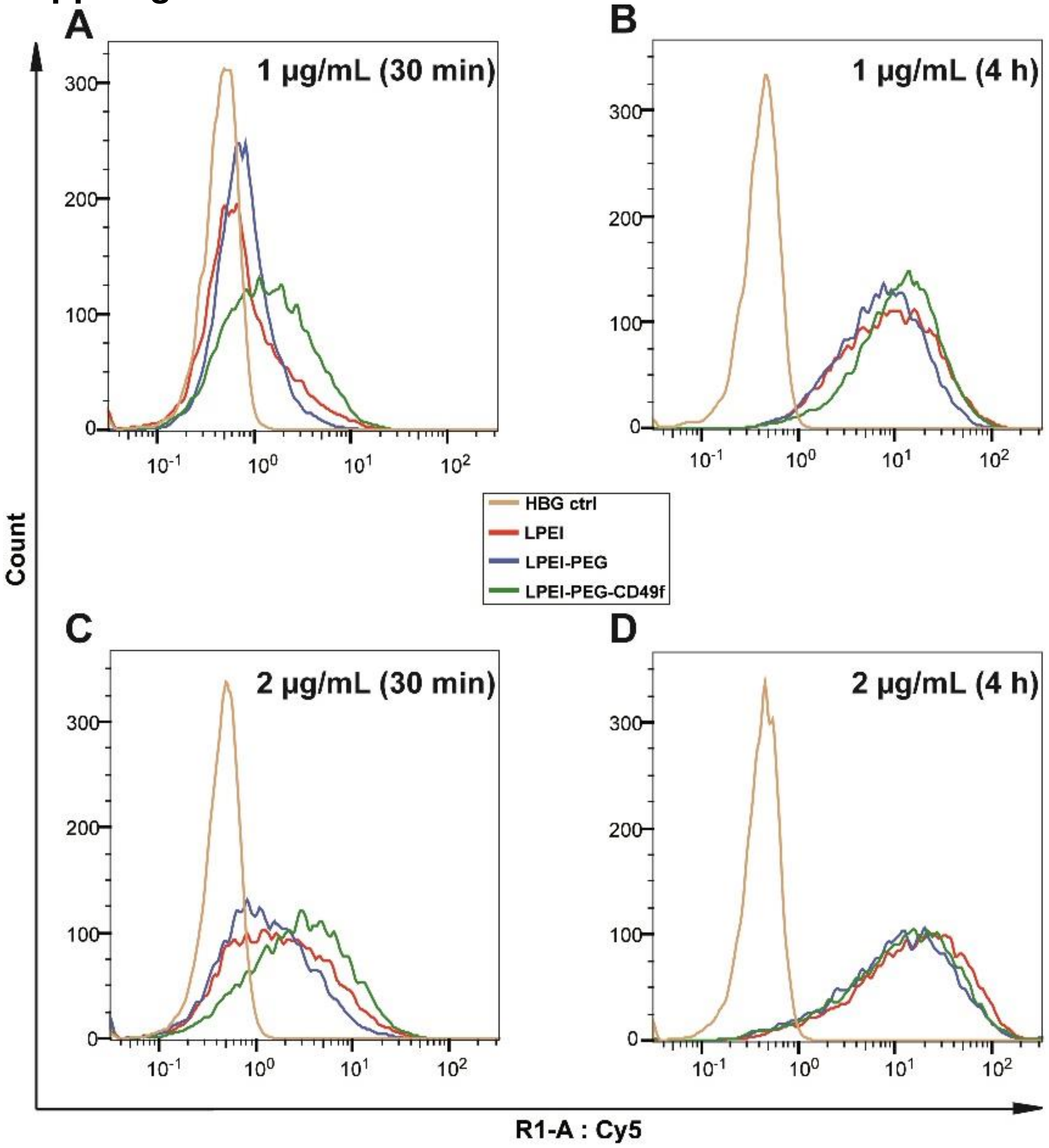

Supp. Figure 2: Cell association of polyplexes. MDA-MB-231 cells were incubated with polyplexes prepared at 20 µg/mL and N/P 9 with Cy5-labelled pDNA diluted in basal medium to a final concentration of 1 µg/mL (A, B) or 2 µg/mL (C,D) for 30 min (A,C) or 4 h (B,D). After incubation, cells were harvested and analyzed by flow cytometry. Data is depicted as representative histograms showing the Cy5 derived fluorescence signal of live cells in the R1 channel (Cy5 fluorescence).

Supp. Figure 3

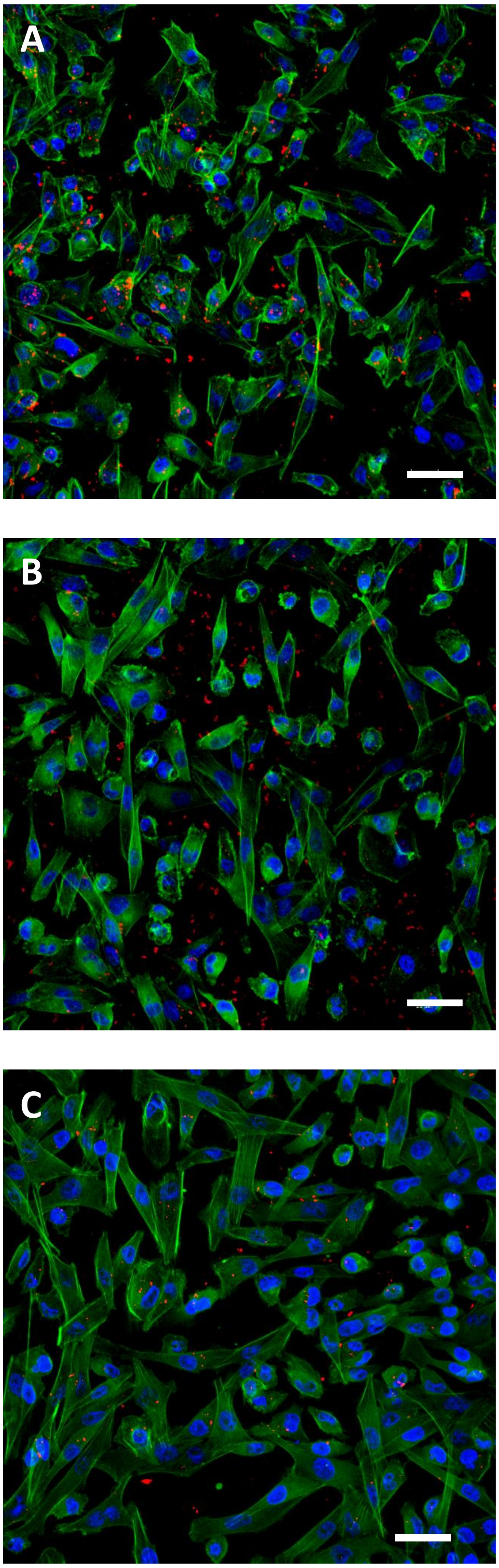

Supp. Figure 3: Cellular association of polyplexes studied by CLSM. MDA-MB-231 cells were incubated in basal cell culture medium with Cy5-labelled polyplexes (red) prepared at N/P 9 at a pDNA concentration of 2 µg/mL for 4 h. Thereafter cells were fixed, stained with AlexaFluor® 488-phalloidin (green) and DAPI (blue). (A) LPEI, (B) LPEI-PEG and (C) LPEI-PEG-CD49f polyplexes. CLSM imaging was conducted with a 20X oil objective; maximum intensity projections of z-sections are shown. Scale bars: 40 µm.

Supp. Figure 4

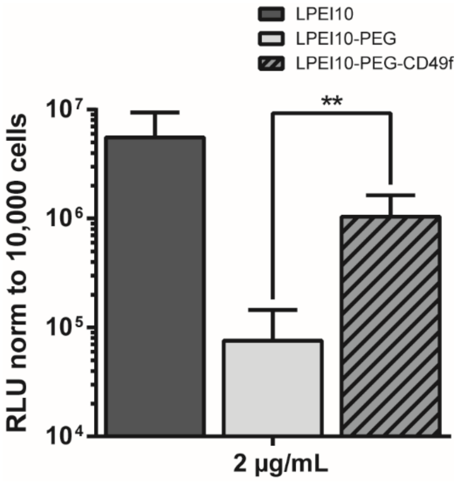

Supp. Figure 4: Transfection study with CT26 cells. CT26 were seeded in 96-well plates and treated with polyplexes based on pCMV-Gluc at 2 µg/mL. 4 h after polyplex addition cellular supernatant was exchanged with cell culture medium supplemented with FCS, L-glutamine and antibiotics. 24 h after starting cell treatment Gaussia luciferase was quantified in the supernatant and RLU values were normalized based on total count of live cells. Data is depicted as mean values (n=3 + stddev; data from 2 independent experiments; ** $p \leq 0.01$ (Mann-Whitney))

Supp. Figure 5

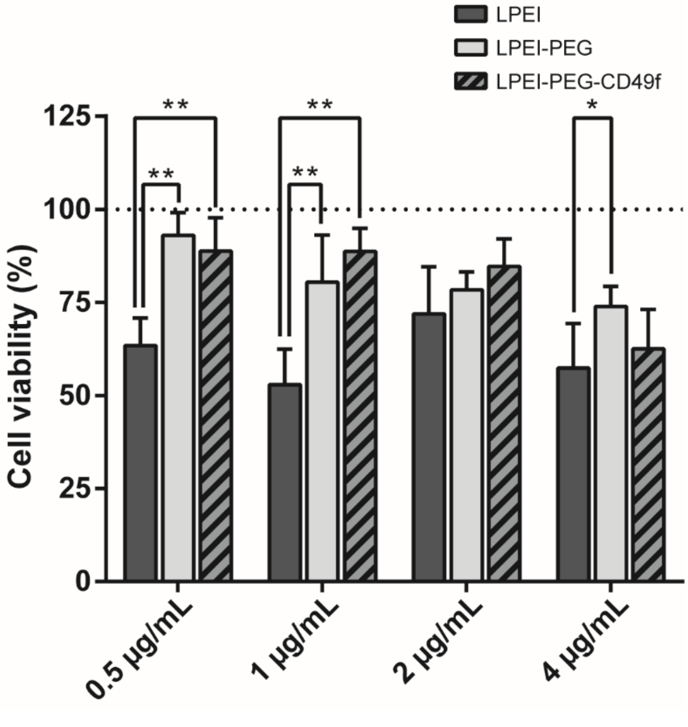

Supp. Figure 5: Cell viability studies by flow cytometry. MDA-MB-231 cells were treated with polyplexes at 4 different concentrations (0.5 µg/mL; 1 µg/mL; 2 µg/mL; 4 µg/mL). 24 h post treatment live cells were counted by flow cytometry after DAPI addition. DAPI derived fluorescence signal was detected in the V1 channel. Total count of live cells was normalized based on buffer treated cells. Data is depicted as mean values (n=3 + stddev; data from 2 independent experiments; *$p \leq 0.05$, ** $p \leq 0.01$ (Mann-Whitney))

Supp. Figure 6

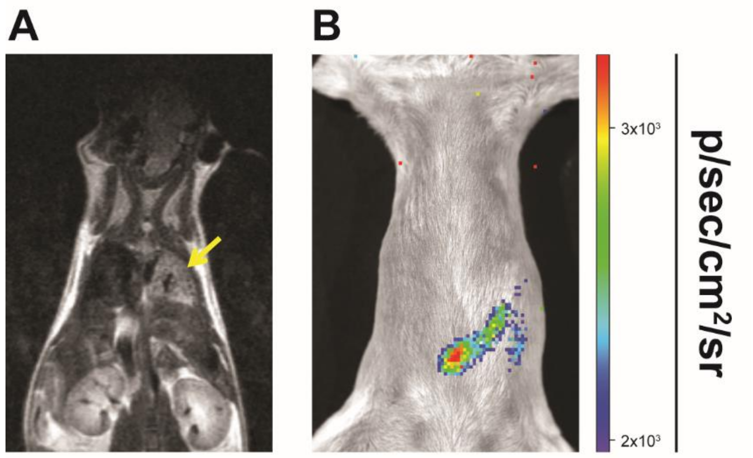

Supp. Figure 6: Representative example of intratracheal polyplex treatment of 4T1-iRFP720 tumor bearing animal with inaccessible tumor tissue. MRI shows intense tumor growth in the whole left lung (marked with yellow arrow) (A). Treatment with LPEI-PEG-CD49f based polyplexes of this animal did not result in sufficient transfection (B). Background BLI signal (color coded) in the abdominal region is depicted as overlay on a reflected light picture.

Supplementary videos:

Video S1:
MRI (T2) coronal fly-through, tumor free control animal shown in Figure 6A

Video S2:
MRI (T2) coronal fly-through, 4T1 tumor bearing animal shown in Figure 6B and Figure 7G

Video S3:
MRI (T2) coronal fly-through, 4T1 tumor bearing animal shown in Figure 7A

Video S4:
MRI (T2) coronal fly-through, 4T1 tumor bearing animal shown in Figure 7C

Video S5:
MRI (T2) coronal fly-through, 4T1 tumor bearing animal shown in Figure 7E

Video S6:
MRI (T2) coronal fly-through, 4T1 tumor bearing animal shown in Figure 7I

Video S7
MRI (T2) coronal fly-through, 4T1 tumor bearing animal shown in Figure 7K

Video S8:
MRI (T2) coronal fly-through, 4T1 tumor bearing animal shown in Supp. Figure 6A

Supplemental methods

Synthesis of linear polyethylenimine (LPEI; Mw 10 kDa)

α-Methyl ω-hydroxy LPEI (Mw 10 kDa) was generated based on a previously described protocol.[1] 2 g α-Methyl ω-hydroxy poly(2-ethyl-2-oxazoline) was dissolved in 50 mL HCl (7 M) and heated overnight under reflux. LPEI precipitated as HCl salt and was purified by centrifugation with HCl (7 M). After dissolving the precipitate in 30 mL water the solution was again heated under reflux and pH was adjusted to 12 with NaOH (1 M). The mixture was cooled to room temperature where LPEI formed a white precipitate as free base. The precipitate was then washed by centrifugation with NaOH (1 M) and water. After resuspending in water the product was lyophilized. Analysis of both structure and purity of LPEI was conducted by ^{1}H-NMR on a Bruker Avance (200 MHz; US) system. Therefore, 5 mg of the product were dissolved in $CDCl_3$. The peak derived by the solvent was used as reference (δ [chemical shift]=7.24 ppm) for evaluation of signals derived from C**H**$_2$-C**H**$_2$-NH and CH_2-CH_2-N**H**. α-Methyl ω-hydroxy poly(2-ethyl-2-oxazoline) was used as control compound for showing complete hydrolysis. GPC analysis was conducted with a GPC/HPLC system equipped with a Catsec-300 column, a UV-Vis and a multiangle light scattering DAWN EOS (Wyatt; Germany) detector. GPC was done with 0.1 % TFA at a flow rate of 0.2 mL/min. For preparing stock solutions used for conjugate synthesis or polyplex generation LPEI was resuspended in water and pH was set to 7.4 with HCl and NaOH. The resulting product was then filtered through a 0.2 µm cellulose acetate membrane. Concentration of LPEI content in aqueous solutions was determined by $CuSO_4$ assay based on UV/Vis spectrophotometry like described elsewhere.[2] For long term storage LPEI was either stored as lyophilized powder under dry conditions at room temperature or dissolved in water at pH 7.4 at -80 °C.

Peptide synthesis

The peptide with the amino acid sequence CYESIKVAVS was synthesized on a semi-automated Biotage Initiator+ system (Sweden). The synthesis setup was based on using Fmoc protected L-amino acids and a ChemMatrix® resin functionalized with Rink-amide linker as solid phase. For every coupling step amino acids were C-terminally activated with 1-hydroxybenzotriazole hydrate (HOBt; 1 eq based on amino acid) and 2-(1H-Benzotriazole-1-yl)-1,1,3,3-tetramethylaminium tetrafluoroborate (TBTU; 1 eq based on amino acid) under addition of N,N-Diisopropylethylamine (DIPEA; 2 eq based on amino acid). The C-terminal amino acid (Fmoc-L-Serin-tBu) was used in a five-fold excess based on the binding capacity of the resin. All following activated amino acids were used in a 2.5-fold excess. Coupling steps of all activated amino acids except activated Fmoc-L-cysteine-Trt were conducted at a temperature of 75 °C for 10 minutes. Coupling of activated Fmoc-L-cysteine-Trt was done twice at 45 °C for 10 minutes. For detection of free terminal primary amino groups Kaiser (ninhydrin) test was conducted after each coupling step.[3] Deprotection of the N-terminus of the growing peptide chain was conducted with piperidine (20 % (V/V) in DMF) twice for 3 minutes at a temperature of 75 °C. Cleavage of the peptide from the resin was done with trifluoracetic acid (TFA):phenol:water:triisopropylsilane (88:5:5:2) for 4 hours at room temperature under vigorous mixing. The solution was then concentrated under vacuum and the product was precipitated with cold diethyl ether. After washing the precipitate with diethyl ether by centrifugation the product was redissolved in water and lyophilized. Purification was conducted by reversed phase chromatography on a Shimadzu HPLC system (Austria) equipped with a Zorbax® 5B-C18 column (21.2 mm x 25 cm). For purification a linear gradient with an increasing concentration of acetonitrile (acidified with 0.1 % TFA) in water (acidified with 0.1 % TFA) starting from 5 to 50 % over 30 minutes at a flow rate of 21 mL/min was chosen. Detection was done at a wavelength of 215 nm. Fractions containing CYESIKVAVS were pooled and lyophilized. Peptide purity was analyzed both by HPLC and high resolution mass spectrometry (HRMS). The analytical HPLC system was equipped with a Thermoquest hypersil division C18 column (Thermo Fisher scientific; Germany) and the same gradient as described before at a flow rate of 1 mL/min was used. HRMS was employed for analysis of purity and amino acid sequence at a sample concentration of 10 µg/mL in 50 % (V/V) ACN (in 0.1 % (V/V) formic acid) with a Bruker maXis Hd system (US). Peptide sequencing was conducted based on single and double charged b- and y-fragments. For further procedure only CYESIKVAVS with a purity of >95 % (based on HPLC) was used.

Supplemental References

1. Rödl, W, Schaffert, D, Wagner, E, and Ogris, M (2013). Synthesis of polyethylenimine-based nanocarriers for systemic tumor targeting of nucleic acids. *Methods Mol Biol* **948**: 105-120.

2. Ungaro, F, De Rosa, G, Miro, A, and Quaglia, F (2003). Spectrophotometric determination of polyethylenimine in the presence of an oligonucleotide for the characterization of controlled release formulations. *J Pharm Biomed Anal* **31**: 143-149.

3. Kaiser, E, Colescott, RL, Bossinger, CD, and Cook, PI (1970). Color test for detection of free terminal amino groups in the solid-phase synthesis of peptides. *Analytical Biochemistry* **34**: 595-598.
