# Supplementary material for: Peptide-Targeted Polyplexes for Aerosol-Mediated Gene Delivery to CD49f-Overexpressing Tumor Lesions in Lung
Source: Mol Ther Nucleic Acids. 2019 Oct 18;18:774–86. doi: 10.1016/j.omtn.2019.10.009 (PMC6861568; doi:10.1016/j.omtn.2019.10.009)
Supplement: Document S2. Article plus Supplemental Information [file mmc10.pdf]

Molecular Therapy
Nucleic Acids
Original Article

Peptide-Targeted Polyplexes for Aerosol-Mediated Gene Delivery to CD49f-Overexpressing Tumor Lesions in Lung

Alexander Taschauer,[1] Wolfram Polzer,[1] Fatih Alioglu,[1] Magdalena Billerhart,[1] Simon Decker,[1] Theresa Kittelmann,[1] Emanuela Geppl,[1] Salma Elmenofi,[1] Martin Zehl,[2] Ernst Urban,[3] Haider Sami,[1] and Manfred Ogris[1]

[1]Laboratory of MacroMolecular Cancer Therapeutics (MMCT), Center of Pharmaceutical Sciences, Department of Pharmaceutical Chemistry, University of Vienna, Althanstrasse 14, A-1090 Vienna, Austria; [2]Faculty of Chemistry, Department of Analytical Chemistry, University of Vienna, Währingerstrasse 38, 1090 Vienna, Austria; [3]Department of Pharmaceutical Chemistry, Faculty of Life Sciences, University of Vienna, Althanstrasse 14, 1090 Vienna, Austria

Peptide ligands can enhance delivery of nucleic acid-loaded nanoparticles to tumors by promoting their cell binding and internalization. Lung tumor lesions accessible from the alveolar side can be transfected, in principle, using gene vectors delivered as an aerosol. The cell surface marker CD49f (Integrin α6) is frequently upregulated in metastasizing, highly aggressive tumors. In this study, we utilize a CD49f binding peptide coupled to linear polyethylenimine (LPEI) promoting gene delivery into CD49f-overexpressing tumor cells *in vitro* and into lung lesions *in vivo*. We have synthesized a molecular conjugate based on LPEI covalently attached to the CD49f binding peptide CYESIKVAVS via a polyethylene glycol (PEG) spacer. Particles formed with plasmid DNA were small (<200 nm) and could be aerosolized without causing major aggregation or particle loss. *In vitro*, CD49f targeting significantly improved plasmid uptake and reporter gene expression on both human and murine tumor cell lines. For evaluation *in vivo*, localization and morphology of 4T1 murine triple-negative breast cancer tumor lesions in the lung of syngeneic BALB/c mice were identified by MRI. Polyplexes applied via intratracheal aerosolization were well tolerated and resulted in measurable transgene activity of the reporter gene firefly luciferase in tumor areas by bioluminescence imaging (BLI). Transfectability of tumors correlated with their accessibility for the aerosol. With CD49f-targeted polyplexes, luciferase activity was considerably increased and was restricted to the tumor area.

INTRODUCTION

Receptor-mediated delivery of nucleic acids to tumor cells is an important issue in the development of viral and synthetic gene delivery systems. Besides increased accumulation in tumors when delivered systemically, their improved internalization into target cells via transfection-permissive uptake routes allows to significantly increase transfection rates and transgene expression. We and others have developed targeted delivery systems based on polycations for nucleic acid condensation, PEG for shielding purposes, and peptidic ligands for receptor targeting.[1–4] Although the affinity of a rather small peptide can be considerably lower when compared with the whole protein, the high number of targeting peptides per transfection particle induces a cooperative binding mode.[5] When compared with their protein counterparts, such peptide ligands are advantageous by several means, i.e., being fully synthetic, less immunogenic, and preferred for the nucleic acid condensation process due to their lower molecular weight.[1] Most of the potentially targetable antigens on tumor cells are not unique *de novo* antigens, but rather overexpressed compared with the surrounding non-malignant tissue.[6] Integrin α6 (ITGA6, CD49f) is a glycosylated transmembrane protein that dimerizes preferentially with integrin β1 and β4 forming laminin binding heterodimers. It is expressed at low to medium levels on a broad range of human tissues, including stomach, intestine, and kidney.[7] CD49f is expressed on almost all tissue stem cell populations including cancer stem cells (CSC)[8] and has recently been described as a key signaling molecule in the maintenance and development of bone mesenchymal stem cells.[9] It is also associated with phosphatidylinositol 3-kinase (PI3K)/Akt signaling and is responsible for maintaining stemness properties in other stem cells by interaction with OCT4 and SOX2.[10] Both benign and malignant cells derived from the prostate capable of spheroid formation show a high CD49f expression, whereas other stem cell markers such as CD133 seem to be less important.[11] In numerous studies it was demonstrated that CD49f-overexpressing tumor cells show tumor-initiating and metastasis-forming potential in numerous solid cancers, including hemangioma,[12] gastric cancer,[13] and breast cancer.[14] In breast cancer patients, CD49f-positive tumors have a dismal clinical prognosis.[15] Also, in murine tumors, CD49f-high cells show tumor-initiating properties[16] and formation of metastasis.[17] The murine

Received 1 June 2019; accepted 12 October 2019;
https://doi.org/10.1016/j.omtn.2019.10.009.

Correspondence: Manfred Ogris, Laboratory of MacroMolecular Cancer Therapeutics (MMCT), Center of Pharmaceutical Sciences, Department of Pharmaceutical Chemistry, University of Vienna, Althanstrasse 14, A-1090 Vienna, Austria.
E-mail: m.ogris@univie.ac.at
Correspondence: Haider Sami, Laboratory of MacroMolecular Cancer Therapeutics (MMCT), Center of Pharmaceutical Sciences, Department of Pharmaceutical Chemistry, University of Vienna, Althanstrasse 14, A-1090 Vienna, Austria.
E-mail: haider.sami@univie.ac.at

 © 2019 The Author(s).
This is an open access article under the CC BY license (http://creativecommons.org/licenses/by/4.0/).

triple-negative breast cancer cell line 4T1 is a highly metastatic and aggressive cancer growing syngeneic in BALB/c mice forming lung metastasis accessible from the alveolar side.[18,19] Stable knockdown of CD49f in 4T1 reduced proliferation and migration *in vitro*.[17] Hence CD49f represents a valuable target for cancer therapeutics including nucleic acid-based drugs (NABDs). The small laminin peptide sequence IKVAV derived from the A chain of laminin has been initially identified by Tashiro et al.[20] for being responsible for cell interaction in a broad range of species, including humans, mice, and rats. The sequence (expanded to SIKVAV) was later on identified to bind to α3, α6, and β1 integrins.[21] Stevenson et al.[22] were the first to utilize this peptide as YESIKVAVS for targeting of an adenoviral vector to α6β1-overexpressing tumor cells both *in vitro* and *in vivo*.

In this study, we show the transfection-promoting properties of CD49f peptide-targeted polymeric gene vectors both *in vitro* and in a syngeneic lung metastasis model in mice through intratracheal aerosolization of polyplex formulations.

RESULTS

Synthesis of LPEI-PEG-Peptide Conjugate

LPEI was synthesized by acidic hydrolysis of poly(2-ethyl-2-oxazoline) with HCl (6 M). Complete cleavage of the side chains of poly(2-ethyl-2-oxazoline) was checked by ^{1}H-NMR where no remaining signals derived from the side chain of the polymer were found. Gel permeation chromatography (GPC) analysis of LPEI revealed an average molecular weight of 10 kDa and a polydispersity (Mw [weight average molecular weight]/Mn [number average molecular weight]) of 1.55.

The peptide CYESIKVAVS was generated with an N-terminal L-cysteine by microwave-assisted solid-phase synthesis based on Fmoc strategy. A ChemMatrix resin functionalized with a Rink amide linker was used to generate a C-terminal carboxamide functionality. The crude product was purified by reversed-phase high-pressure liquid chromatography (HPLC) with a linear gradient from 5% to 50% acetonitrile (ACN), resulting in a product purity of >95%. High-resolution mass spectrometry (HRMS) of the intact peptide, as well as extensive series of collision-induced dissociation (CID)-generated single- and double-charged y- and b-fragment ions, confirmed both purity and the desired sequence CYESIKVAVS.

Conjugate synthesis and analysis were done in principle as described previously.[1,23] In brief, CYESIKVAVS was coupled to LPEI via a heterobifunctional PEG (MW 2 kDa) linker with α-OPSS (3-(2-pyridyldithio)propionamide) and ω-NHS (*N*-hydroxysuccinimide) functionality. ^{1}H-NMR of the intermediate compound, LPEI-PEG-OPSS, revealed a LPEI/PEG ratio of 1:1.2. A solution of LPEI-PEG-OPSS in water with a concentration of 2 mg/mL showed an OPSS concentration of 84.65 nmol/mL. A 3-fold molar amount of peptide was used for generating LPEI-PEG-CYESIKVAVS. Due to the weak absorption of CYESIKVAVS at 280 nm, no direct peptide quantification could be conducted in the conjugate. Nevertheless, near-quantitative reaction of the free thiol group in CYESIKVAVS with α-OPSS was observed when measuring the increase in absorption at 343 nm generated by 2-thiopyridone released during the coupling reaction (ε = 8,080 M^{-1}). Therefore, it was assumed that 1.2 mol PEG per LPEI molecule calculated by ^{1}H-NMR (described above) carries 1.2 mol peptide. The synthesized conjugate is later termed LPEI-PEG-CD49f. For non-targeted formulation, LPEI-PEG-cysteine (LPEI-PEG) conjugate was employed.

Nanoparticle Tracking Analysis

Polyplex nanoparticles were prepared by complexing plasmid DNA with LPEI-PEG-CD49f or LPEI-PEG at nitrogen/phosphate (N/P) 9 to form peptide-targeted or non-targeted formulations, respectively. Only LPEI-based polyplexes served as control for PEGylated formulation. Nanoparticles were analyzed by nanoparticle tracking analysis (NTA) to determine the influence of PEGylation on size and ζ-potential of polyplexes, and potential aggregation effects induced by microspraying (Figure 1).

At 20 μg/mL, mean particle size of polyplexes based on both PEGylated formulations (LPEI-PEG; LPEI-PEG-CD49f) was approximately 30% below the average size of LPEI polyplexes (Figure 1A) (LPEI: 113 ± 8 nm, LPEI-PEG: 75 ± 1 nm, LPEI-PEG-CD49f: 81 ± 2 nm). At 267 μg/mL, an increase of particle size of 6%–14% with PEGylated conjugates could be observed in comparison with LPEI polyplexes (LPEI: 144 ± 4 nm, LPEI-PEG: 160 ± 10 nm, LPEI-PEG-CD49f: 154 ± 3 nm). When generating polyplexes at 267 μg/mL, the fraction of particles below 200 nm in size was reduced by approximately 20% when compared with their respective counterparts generated at 20 μg/mL pDNA (Figure 1B).

LPEI-based polyplexes had a ζ-potential of 35.6 ± 1.01 mV, whereas with LPEI-PEG and LPEI-PEG-CD49f polyplexes this value was reduced (22.6 ± 0.50 mV for LPEI-PEG, 28.4 ± 0.98 mV for LPEI-PEG-CD49f) (Figure 1C). The influence of microspraying on particle size and aggregation effects was analyzed for polyplexes generated at 267 μg/mL (Figures 1D–1F; Figure S1). For all three formulations (LPEI, LPEI-PEG, and LPEI-PEG-CD49f), a slight reduction of nanoparticle concentration after microspraying for sizes up to 400 nm occurred (Figures 1D–1F). LPEI-based polyplexes showed only a minor increase of mean particle size from 131 ± 1 to 143 ± 3 nm after the microspraying procedure (Figure S1A). In case of LPEI-PEG, a change of particle size from 154 ± 1 to 169 ± 4 nm and LPEI-PEG-CD49f from 148 ± 5 to 163 ± 5 nm could be observed. Overall, a reduction of the percentage of particles below a size of 200 nm of 4% for LPEI, 10% for LPEI-PEG, and 8% for LPEI-PEG-CD49f could be detected (Figure S1B).

Cellular Binding and Uptake of Targeted Polyplexes on CD49f-Expressing Cells

Cell binding and internalization of fluorescently labeled polyplexes were evaluated on CD49f-overexpressing cells *in vitro* by flow cytometry and confocal laser scanning microscopy (CLSM). First, CD49f expression was verified on human (MDA-MB-231) and murine (CT26 and 4T1-iRFP720) cancer cell lines by flow cytometry (Figure 2). For all three cell lines a shift of 1–2 log units could be observed

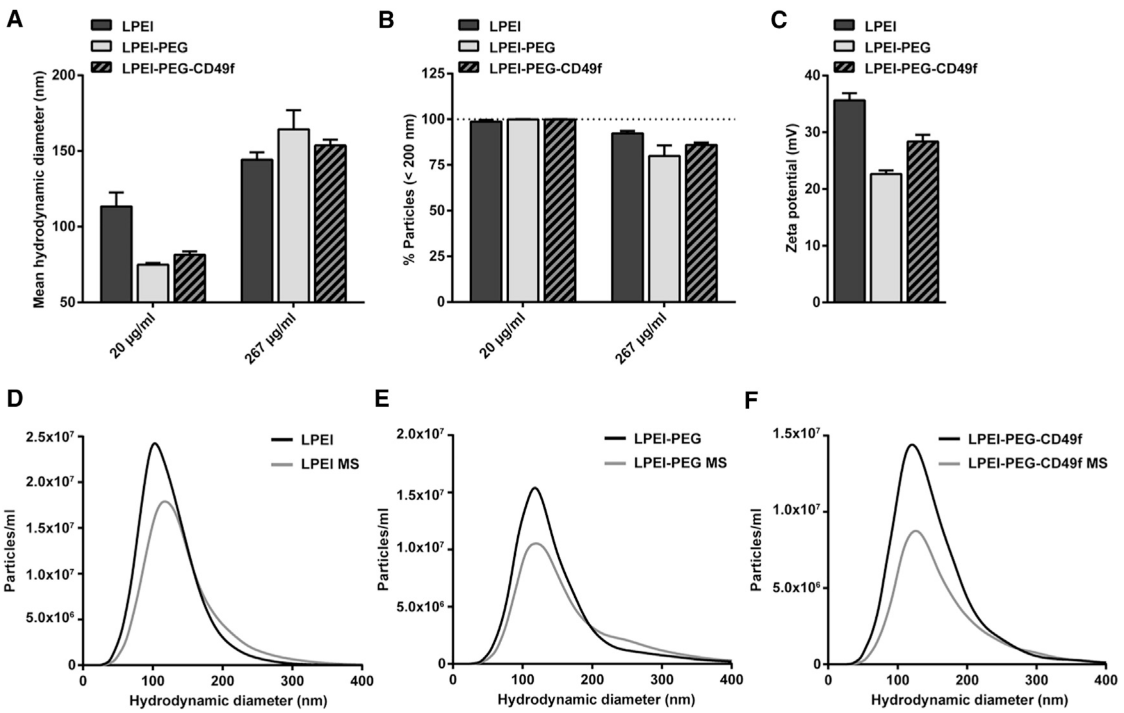

Figure 1. Size and ζ-potential of Polyplexes Evaluated by NTA

(A and B) Hydrodynamic diameter (A) and changes in the percentage of particles below 200 nm (B) of polyplexes prepared at pDNA concentrations of 20 or 267 μg/mL at N/P 9 in HBG. (C) ζ-Potential of polyplexes generated with 267 μg/mL pDNA at N/P 9 (n = 3 + SD). (D–F) Representative particle size distribution of polyplexes based on LPEI (D), LPEI-PEG (E), and LPEI-PEG-CD49f (F) at pDNA concentration of 267 μg/mL and N/P 9 before and after aerosolization by microspraying (MS).

indicating high CD49f expression for MDA-MB-231 (Figure 2A) and medium for CT26 (Figure 2B) and 4T1-iRFP720 (Figure 2C). Geometric mean values of anti-CD49f-stained cells were 43.3 for MDA-MB-231, and 10.8 and 11.7 for CT26 and 4T1-iRFP720, respectively (Figure 2D).

Evaluation of cell binding and uptake was conducted on MDA-MB-231 cells utilizing polyplexes with Cy5-labeled plasmid pCMV-Gluc and analyzed by flow cytometry (Figure 3; Figure S2). At the 30-min time point, LPEI-PEG-CD49f polyplexes exhibited significantly increased cell binding when compared with LPEI-PEG at both concentrations evaluated, whereas only at 2 μg/mL of the signal measured with LPEI-PEG polyplexes was lower when compared with LPEI polyplexes. At 4-h incubation with 1 μg/mL, there was no gross difference observed between all three polyplex types, whereas with 2 μg/mL both PEG-containing polyplexes exhibited lower total cell association when compared with LPEI polyplexes (Figure 3).

In order to demonstrate cellular association and the intracellular localization of polyplexes, CLSM studies were conducted after 4 h of incubation at 2 μg/mL (Figure 4; Figure S3). Co-staining of actin filaments with Alexa Fluor 488-phalloidin enabled localization of particles both on the cellular surface and intracellularly, when visualizing the middle sections of cells. First, samples were analyzed using a 20× objective recording a z stack ranging from the plastic surface to the top of the cells (Figure S3) followed by maximum intensity projection. With LPEI polyplexes, larger aggregates were observed either associated with the plastic surface or the cells. LPEI-PEG polyplexes exhibited a similar distribution pattern, but with overall lower cellular association. Similarly, with LPEI-PEG-CD49f polyplexes, total cellular association was lower when compared with LPEI polyplexes. With higher magnification (63×), central sections of cells were analyzed for internalization status of polyplexes. Here, LPEI particles were internalized to a considerable extent (Figure 4A), but less internalized LPEI-PEG-based particles were observed (Figure 4B). Employing LPEI-PEG-CD49f conjugate for polyplex generation resulted in an enhanced cellular uptake of particles (Figure 4C).

Targeted Gene Delivery and Biocompatibility Profile

For evaluating *in vitro* transfection efficiency, polyplexes were generated with plasmid pCMV-Gluc. Because Gaussia luciferase (Gluc) is secreted by the cells, we quantified its activity in the cellular supernatant by a bioluminescence assay using coelenterazine as substrate. In the case of MDA-MB-231 and CT26 cells, remaining cells were harvested and cell numbers determined by flow cytometry. Total count of live cells was used for normalization of bioluminescence signal. In the case of 4T1-iRFP720 cells, Gaussia activity was also measured in the supernatant, but its activity had to be normalized on protein content per well quantified by bicinchoninic acid (BCA) assay, because it was not possible to obtain a single-cell suspension and to quantitatively

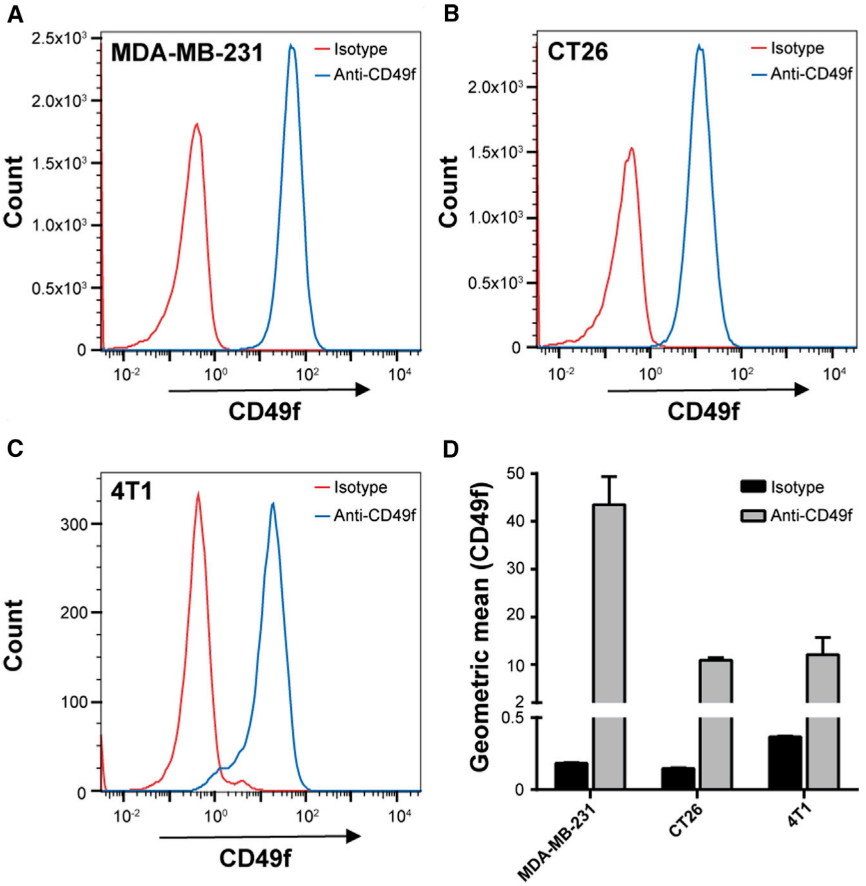

Figure 2. Evaluation of CD49f Expression by Flow Cytometry

MDA-MB-231, CT26, and 4T1-iRFP720 cells were harvested, stained with BV421-labeled rat anti-human CD49f antibody or rat IgG2a κ isotype control, and gated cells were analyzed. (A–C) Representative histograms of MDA-MB-231 (A), CT26 (B), and 4T1-iRFP720 (C). (D) Geometric mean values for BV421 signal (n = 3 + SD).

remove cells from the well surface. MDA-MB-231 and 4T1-iRFP720 cells were transfected with polyplexes generated at N/P 9 at pDNA concentrations of 0.5, 1, 2, and 4 μg/mL (Figures 5A and 5B). In the case of LPEI-PEG-CD49f, this corresponds to a peptide concentration of 70.4, 140.7, 281.5, and 562.9 nM, respectively. Transfection studies with CT26 were conducted with 2 μg/mL (Figure S4). For all three cell lines, Gaussia reporter gene activity obtained with LPEI-PEG polyplexes was at least 10-fold lower when compared with plain LPEI polyplexes. Also, at all concentrations evaluated and with all three cell types, except 1 μg/mL with MDA-MB-231 cells, a significant increase of transfection efficiency when using LPEI-PEG-CD49f conjugate could be observed compared with LPEI-PEG. On MDA-MB-231 cells, the increase was 2.3× (2.5 μg/mL), 11.1× (1 μg/mL), 9.6× (2 μg/mL), and 4.3× (4 μg/mL), whereas on 4T1-iRFP720 cells, the increase was 2.5× (0.5 μg/mL), 10.6× (1 μg/mL), 5.5× (2 μg/mL), and 6.4× (4 μg/mL), and on CT26 the increase was 13.7× (2 μg/mL). Cell viability was evaluated on MDA-MB-231 with flow cytometry quantifying viable cells per well with buffer-treated ones set to 100% along with transfection efficiency (Figure S5). At all polyplex concentrations used, LPEI polyplex treatment resulted in the most prominent reduction of viable cells (53% to 72% compared with untreated control). Both LPEI-PEG-CD49f and LPEI-PEG polyplex treatments exhibited a significantly higher cell viability at concentrations of 0.5 and 1 μg/mL. At 4 μg/mL, cell viability was significantly higher for LPEI-PEG polyplex treatment when compared with LPEI-polyplex-treated cells.

Tumor Model

The tumor model was established by intravenous injection of 4T1-iRFP720 cells as recently described,[19] and tumor growth was monitored by T2-weighted MRI (Figure 6; Videos S1 and S2). On all T2-weighted MRI images, tumor tissue could be visualized as a hyperintense structure. A clear differentiation between tumor tissue and blood vessels could be made based on the comparison with untreated control animals (Figures 6A and 6B). Whereas larger blood vessels in the lung appeared as hyperintense structures, lung parenchyma of tumor-free animal exhibited an overall homogenously hypointense area. At 11 days post tumor cell implantation, tumor nodules appeared as hyperintense areas at various regions in the lung (Figure 6B). Lung histopathology confirmed the findings by Geyer et al.[19] revealing both peribronchiolar and parenchymal tumor tissue with invasive growth pattern and accessibility from the alveolar side (Figures 6C and 6D). Immunohistochemistry could prove CD49f overexpression of 4T1-iRFP720 tumors (Figures 6D and 6E), but also a lower expression level in healthy lung tissue.

Aerosol Mediated Gene Delivery to Lung Tumor Lesions

Before transfection, tumor localization was analyzed by T2-weighted MRI (Figure 7A and Video S3, Figure 7C and Video S4, Figure 7E and Video S5, Figure 7G and Video S2, Figure 7I and Video S6, Figure 7K and Video S7). Both the formation of a rather large, homogenously structured, well-margined tumor tissue (Figures 7A, 7C, and 7G) and multiple small heterogeneous lesions (Figures 7E, 7I, and 7K) could be found. Such diffuse lesions were sometimes located along the tracheal bifurcation with infiltration into the bronchial airways (Figures 7E and 7K). Animals were treated by microspray-based aerosolization of polyplexes on day 11 after tumor cell implantation. Twenty-four hours post transfection, luciferase activity was evaluated by 2D BLI (Figures 7B, 7D, 7F, 7H, 7J, and 7L). With both treatments, either LPEI-PEG-CD49f polyplexes (Figures 7B, 7F, and 7J) or LPEI-PEG polyplexes (Figures 7D, 7H, and 7L), distinct BLI signals could

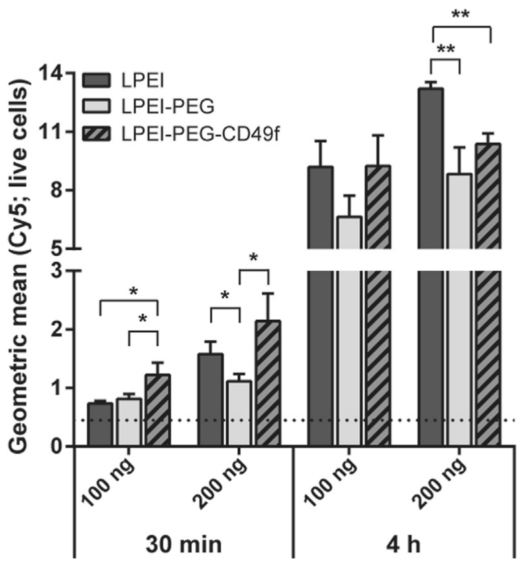

Figure 3. Mean Cellular Association of Polyplexes

MDA-MB-231 cells were incubated with polyplexes based on Cy5-labeled pDNA prepared at N/P 9. Cells were treated in basal cell culture medium with two different concentrations and durations (1 μg/mL for 30 min and 4 h; 2 μg/mL for 30 min and 4 h), analyzed by flow cytometry, and the geometric mean values were calculated. Data are depicted as average value from three independent samples with 1×10^4 per sample (n = 3 + SD; *p < 0 0.05; **p < 0.01; t-test, two-tailed). The background signal is shown as a dotted line.

be detected in the lung area. Of note, areas of maximal intensity are on average 10-fold higher in animals transfected with PEG-CD49f polyplexes when compared with animals transfected with LPEI-PEG polyplexes (see scale bars in Figures 7B, 7F, and 7J versus Figures 7D, 7H, and 7L). When placing BLI images and MRI images of the same animal side by side, there was high degree of correlation between the tumor localization depicted by MRI and the site of the BLI signal. In Figures 7A and 7B (and also in Figures 7I and 7J), both the hyperintense MRI signal and the BLI signal are found in the upper left lung. The MRI signal in Figure 7A is of diffuse nature suggesting parenchymal tumor infiltration, whereas in Figure 7I the tumor appears nodular. In Figure 7F, a strong BLI signal is seen in the right lung and a weaker one in the left lung. The corresponding MRI picture in Figure 7E shows a diffuse lesion in the median and right lung area. In the fly-through video (Video S5), there is also a diffuse lesion visible in the left lung. In Figure 7C, a diffuse parenchymal signal can be observed in the upper right lung, and a more hyperintense lesion in the median area. Both parts appear positive in BLI (Figures 7D). In Figure 7K, both sides of the lungs carry nodular lesions but also parenchymal infiltration (as visible in the fly-through video, Video S7). Apparently, here only the tumor area in the right lung is transfected, as shown in Figure 7L. In animals with higher tumor load and reduced accessibility of lung tissue, application of sufficient volumes of aerosol was not possible, and hence no reporter gene expression was detected after treatment. In the representative example shown (Figure S6; Video S8), the left lung showed a homogeneous growth of tumor lesions, hence reducing accessibility of tumor tissue through the airways. The BLI signal in successfully treated animals was quantified by analyzing the BLI images (Figure 8). Here, BLI signals of animals treated with LPEI-PEG-CD49f polyplexes were significantly higher than background signal, which was not the case with the LPEI-PEG-based formulation.

DISCUSSION

The peptide sequence SIKVAV (IKVAV) has initially been identified as the functional domain within the α chain of laminin, which is responsible for cellular interaction.[20] Later on, α3, α6, and β1 integrins were identified as binding partners on the cell surface.[21] Upon binding, the peptide can activate PI3K/Akt downstream signaling and extracellular signal-regulated kinase 1/2 (ERK1/2) and matrix metalloproteinase (MMP) expression,[24] albeit at high concentrations of 0.1 mM and above.[21,25] To achieve high local concentrations, it is included in polymeric carriers or nanogels and applied locally, for example, in wound healing approaches.[26] Our approach here was to use the peptide as a targeting moiety covalently coupled to the nucleic acid carrier LPEI, but at low, non-activating concentrations (<0.6 μM). For this, we have generated a molecular conjugate with equimolar ratios of peptide, a heterobifunctional, 2-kDa PEG linker, which also serves as shielding agent,[1,27] and a 10-kDa LPEI for DNA binding and condensation. Already in our previous work we could demonstrate that coupling of the amine-reactive NHS group in NHS-PEG-OPSS to secondary amines within the LPEI chain is efficient and controllable when carried out in water-free conditions either in DMSO or absolute EtOH, resulting in a coupling efficiency of approximately 60%.[27] The extended peptide sequence CYESIKVAVS was then coupled in a 4-(2-hydroxyethyl) piperazine-1-ethanesulfonic acid (HEPES)-buffered aqueous solution at pH 7.4 to the proximal OPSS group. Whereas the N-terminal cysteine was added to enable thiol coupling, the other residues improve water solubility of the peptide.[28]

A thorough biophysical characterization was conducted to study effects of PEG linker, targeting peptide, higher particle concentrations for *in vivo* application, and the shear stress during the nebulization procedure. For stability reasons, all polyplex formulations were prepared with positive charge excess at N/P 9. This ratio prevents aggregation during polyplex formation also at higher concentrations and is still well tolerated *in vivo*.[19,29] The 2-kDa PEG linker is significantly reducing the ζ-potential, and in our previous studies we could demonstrate that such pDNA polyplexes are stable enough in the bloodstream to transfect tumor tissue after systemic administration.[4,30] No differences were observed in terms of average particle size or aggregation between polyplexes ± PEG after microspraying, indicating that the elevated N/P ratio is already ensuring enough stability.[19]

We could confirm the already observed high level of CD49f in human MDA-MB-231 and murine 4T1 triple-negative breast cancer cells,[17,31] but also demonstrated this in the murine colon carcinoma cell type CT26. Similarly, elevated CD49f expression was also seen in the human tumor cell line A549 adenocarcinoma and an ECV304-derived subline (data not shown). The high level of CD49f

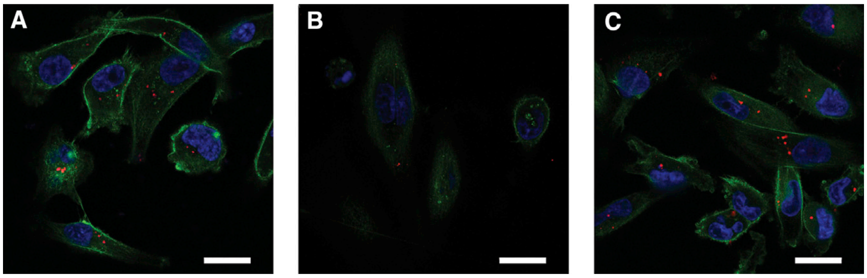

Figure 4. Cellular Internalization of Polyplexes Studied by CLSM

MDA-MB-231 cells were incubated in basal cell culture medium with Cy5-labeled polyplexes (red) prepared at N/P 9 at a pDNA concentration of 2 μg/mL for 4 h. Thereafter cells were fixed and stained with Alexa Fluor 488-phalloidin (green) and DAPI (blue). (A–C) LPEI (A), LPEI-PEG (B), and LPEI-PEG-CD49f (C) polyplexes. CLSM imaging was conducted with a 63× oil objective; representative middle sections of the cells are shown. Scale bars: 20 μm.

expression in MDA-MB-231 cells is in line with previous observations, where in this and other highly malignant breast cancer cell lines, ITGA6 expression was elevated and mediated radio-resistance via the activation of the PI3K/Akt and MEK/Erk signaling pathways.[32]

Binding of polyplexes to the cell surface occurs either via electrostatic interaction, e.g., heparan sulfate proteoglycan binding, or receptor-ligand interaction. We and others have often observed rapid aggregation of LPEI-based polyplexes in cell culture when incubated in serum-free medium, which also influenced the uptake route leading to efficient transgene expression.[33,34] Similar observations were made here with LPEI polyplexes, where particles appear attached to the cell culture plastic, but also the cell surface (Figure S3A). PEGylated polyplexes without ligand exhibited an overall lower cell association (Figure 3; Figures S2A and S2C), which is in line with our previous work using 2 kDa PEG as a shielding moiety.[2] After 4 h of incubation, total cellular association of LPEI-PEG-CD49f was similar to untargeted ones (Figure 3; Figures S2B and S2D), but the internalization rate appeared higher (Figure 4). Although total cell association of positively charged (unshielded) polyplexes and targeted ones (but with decreased positive surface charge) can be similar, they can differ in their binding and internalization kinetics. Polyplexes with epidermal growth factor (EGF) as ligand are rapidly internalized within minutes, which is triggered after activation of downstream signaling (mitogen-activated protein kinase [MAPK], Akt/Erk) followed by membrane ruffling.[1,2,35] In contrast, a receptor binding mode, which does not activate downstream signaling, is similarly efficient, but internalization is delayed and occurs via a rather slow, actin-driven receptor recycling.[2] When comparing cell association (by flow cytometry) and internalization (by CLSM), CYESIKVAVS polyplexes showed higher association rates at earlier time points and similar or slightly lower ones at later time points and higher concentration than LPEI polyplexes (Figure 3). Still, internalization was rather slow, because no significant intracellular signal of LPEI-PEG-CD49f polyplexes was seen with CLSM after 30 min of incubation (data not shown), but rather occurred at the 4-h time point (Figure 4). Hence we do not expect rapid internalization due to pathway activation, like with EGF-EGFR, but rather slow actin-driven receptor-ligand recycling. This would be also in line with the peptide concentrations applied: our highest concentration applied (4 μg/mL pDNA) corresponds to a peptide concentration of 0.563 μM (considering an N/P of 9 and a LPEI-PEG-peptide ratio of 1:1.2:1.2 in the conjugate). This is still far below the concentration of 100 μM needed for Erk and Akt activation.[25] For evaluation of transfection efficiency *in vitro*, we utilized secreted Gluc.[36] At all concentrations and cell lines tested, PEG-polyplexes without ligand reduced reporter activity by 90% or more, whereas the peptide ligand could fully restore activity or induce an even higher activity when compared with "naked" LPEI polyplexes. Of note, there is also a clear difference in the aggregation behavior of "naked" LPEI polyplexes and their PEGylated counterparts *in vitro*: LPEI polyplexes usually rapidly aggregate in serum-free media (or when generated in salt-containing buffer), which boosts transfection efficiency *in vitro*.[33,34] In contrast, already the 2-kDa PEG linker attached to LPEI prevents such aggregation of initially small particles also in serum-free media

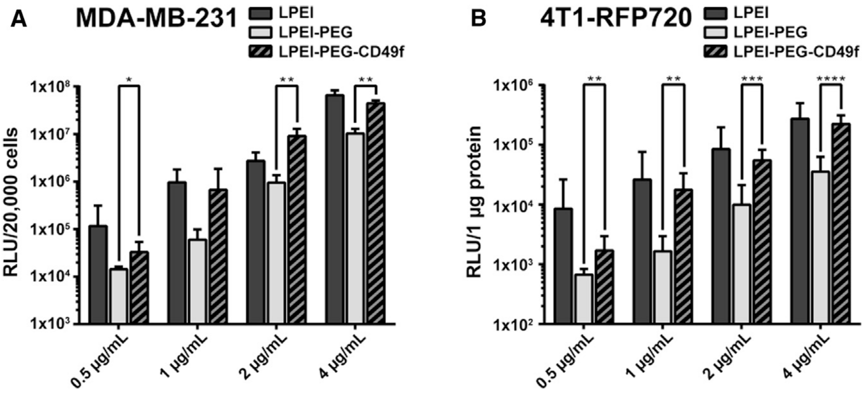

Figure 5. Transfection Efficiency of Polyplexes on MDA-MB-231 and 4T1-iRFP720 Breast Cancer Cells

(A and B) MDA-MB-231 (A) and 4T1-iRFP720 cells (B) were seeded in 96-well plates and transfected with pCMV-Gluc polyplexes at indicated pDNA concentrations. Transfections were conducted in basal cell culture medium for 4 h followed by exchanging the supernatant with fetal calf serum (FCS)-supplemented medium. Gluc was quantified in the supernatant 24 h after transfection, and RLU values were normalized on total count of live cells (MDA-MB-231) or on protein content (4T1-iRFP720). Mean values are from two independent experiments with n = 3/experiment + SD. *p ≤ 0.05, **p ≤ 0.01, ***p ≤ 0.001, ****p ≤ 0.0001 (Mann-Whitney).

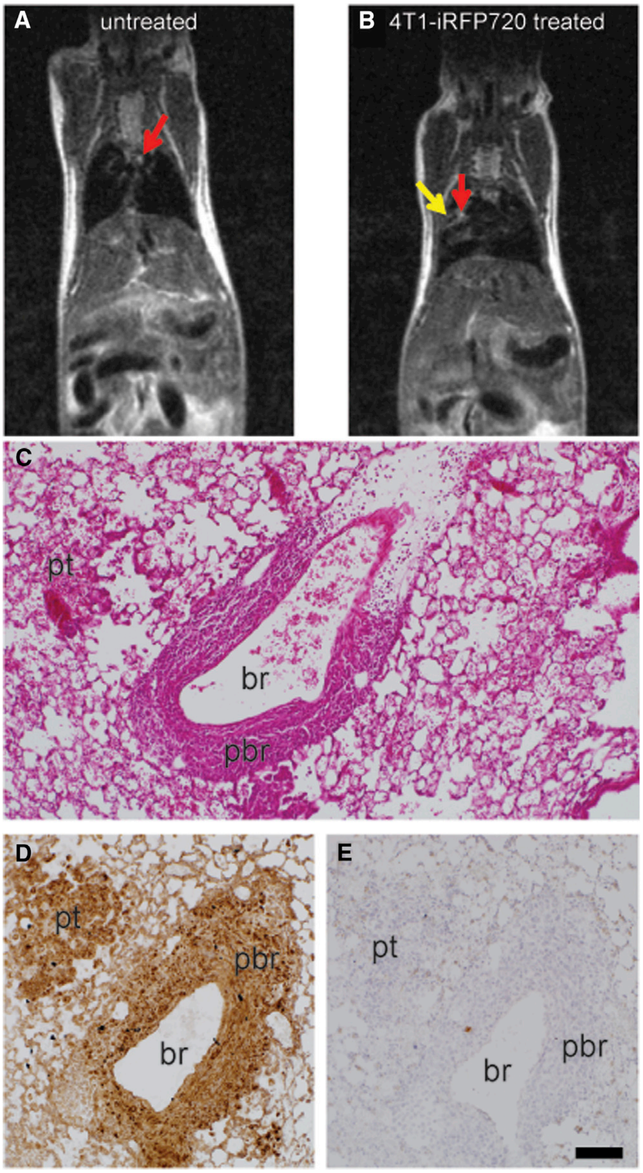

Figure 6. Morphological and Histological Evaluations of the 4T1-iRFP720 Tumor Model

(A and B) Representative T2-weighted MRI images of tumor-free (A) and 4T1-iRFP720 tumor-bearing BALB/c mice (B); day 10 post tumor cell injection. Red arrows: blood vessels; yellow arrow: tumor tissue. (C–E) Histopathology of an instillation fixed lung bearing 4T1-iRFP720 tumors: (C) H&E staining, (D) IHC staining for CD49f (brown), and (E) isotype control antibody. br, lumen of the bronchiole; pbr, peribronchiolar tumor tissue; pt, parenchymal tumor area. Scale bar: 100 μm (C–E).

and reduces their transfection efficiency.[1,2] We did not observe a correlation between the level of CD49f expression (as shown in Figure 2) and the increase in transgene activity comparing PEGylated with targeted polyplexes. Both in MDA-MB-231 and 4T1-iRFP720 cells the increase was comparable (approximately 2.5-fold to >10-fold). This also points out that the 2-kDa PEG linker does shield particles (as demonstrated by the lower ζ-potential), but does not negatively affect the intracellular transfection process, like endosomal release. Especially in case of excessive PEGylation, this can otherwise lead to an inhibitory effect called PEG dilemma.[37] Specificity of the peptide for targeting nucleic acid carriers was already demonstrated by Stevenson et al.[22] using a scrambled version, and due to the slightly acidic isoelectric point of 5.99 (calculated using the online tool ExPASy: https://web.expasy.org/compute_pi/), non-specific, only charged-induced binding is not expected. In the original publication by Tashiro et al.,[20] the IKVAV sequence was described to promote cell adhesion in cells of murine and human origin. The sequence appears in the laminin A chain of both human and murine origin at a similar position (human: position [pos.] 2166–2170, mouse: pos. 2173–2177, source: Universal Protein Resource [UniProt, https://www.uniprot.org]; P25391 [LAMA1_HUMAN], P19137 [LAMA1_MOUSE]). CD49f (Integrin α6, ITGA6) shows a 98.3% similarity between the human and the murine form. Hence we expect a very similar binding behavior of our targeting peptide used with the human and the murine CD49f. This is also reflected in the comparable level of enhanced transfection efficiency in human MDA-MB-231 and murine 4T1-iRFP720 and CT26 cells. Although the CD49f expression was higher in MDA-MB-231 cells, the effect on transfection enhancement was similar in all three cell lines. Thus, it seems that the binding of peptide to both human and murine CD49f is similarly effective. However, one has to be careful in making that conclusion because transfection efficiencies can be influenced by a multitude of factors.

For basal-like and triple-negative breast cancer, the lung is the most common site of metastasis, and there are several studies indicating that the presence of CSCs in triple-negative breast cancer promotes this metastasis formation.[38] Within the syngeneic 4T1 model, lung lesions can be induced either by orthotopic cell implantation and subsequent removal of the primary tumor, or by intravenous injection of the cell suspension.[39] Because with both methods the genomic profile of lung lesions is similar, intravenous injection appears to be a useful and less invasive method. Whereas for the intravenous models using a luciferase-labeled 4T1 subline, tumors were located parenchymally without the appearance of individual lesions, we also observed parenchymal infiltration, but with a clear nodular structure and also peribronchiolar localization using our own iRFP720-labeled subline.[19] Immunohistochemical (IHC) analysis of instillation fixed lungs revealed that both parenchymal-like and peribronchiolar lesions were overexpressing CD49f, and $CD49f^{+}$ cells appeared accessible from the air side being located in the alveolar septa and also inside the alveolar space. In line with the histomorphological analysis, the T2-weighted MRI analysis also reflected the situation of both margin-defined tumor lesions and also diffuse parenchymal infiltrations. Similar correlations could be shown in a model of orthotopically implanted 4T1 tumors followed by lung metastasis: here, mainly well-defined tumor lesions were seen both with histomorphology and T2-weighted MRI.[40]

In addition to low-molecular-weight drugs, also macromolecules, including proteins and nucleic acids and nanostructured carriers,

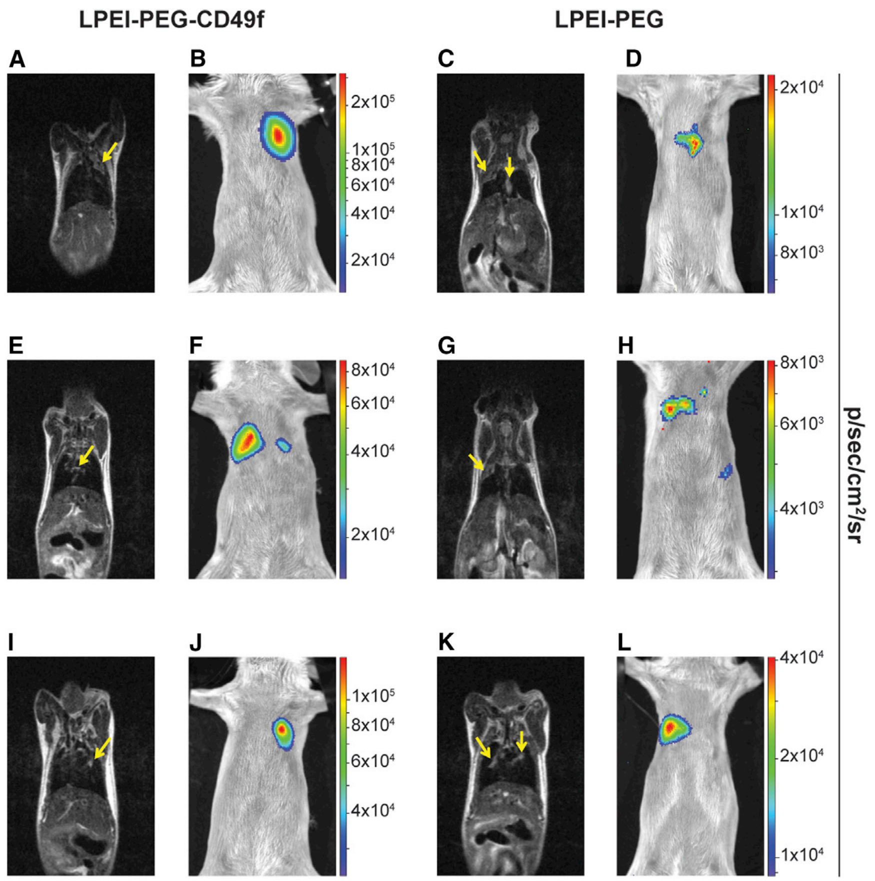

Figure 7. Intratracheal Transfection of 4T1-iRFP720 Tumor-Bearing Lungs

4T1-iRFP720 tumor-bearing BALB/c mice were treated with polyplexes based on pCpG-hCMV-EF1α-LucSH and LPEI-PEG-CD49f or LPEI-PEG at N/P 9 and a concentration of 267 μg/mL. In total, 20 μg of formulated pDNA was administered by intratracheal (i.t.) aerosolization. Twenty-four hours posttreatment, firefly luciferase expression was analyzed by *in vivo* BLI imaging. T2-weighted MRI was conducted 1 or 2 days before treatment for evaluating the status of tumor growth. (A–L) Both BLI and MRI were done in supine positioning of the animal. Three representative animals treated with LPEI-PEG-CD49f (A, E, and I show MRI of animals 1, 2, and 3, respectively; B, F, and J show BLI of animals 1, 2, and 3, respectively) and LPEI-PEG (C, G, and K show MRI of animals 1, 2, and 3, respectively; D, H, and L show BLI of animals 1, 2, and 3, respectively) polyplexes are depicted. Tumor growth is marked with yellow arrows in the MRI images. BLI signals (color coded) are overlaid on a reflected light picture.

can be applied as an aerosol or by instillation to primary and secondary lung tumors.[41] Direct accessibility of the tumor, reduced distribution in non-target tissue, and elevated local drug concentrations are major advantages of this method. Branched polyethylenimine (BPEI) was successfully used for aerosol-mediated plasmid delivery, also in a therapeutic setting.[42,43] We have recently used LPEI and could observe a low, but tumor-restricted, expression of the luciferase reporter gene.[19] Due to the improved transfection *in vitro* and the accessibility of CD49f$^+$ cells, we evaluated both peptide-targeted and PEGylated only polyplexes. Although with PEGylated polyplexes a defined BLI signal could be measured, the signal was low and failed significance when compared with background values when measuring intensities in similar-sized regions of interest (ROIs). Of note, not all tumor areas were transfected when comparing MRI and BLI data side by side, e.g., as seen in Figures 7K and 7L. A comparably low, but locally restricted, BLI signal was observed with similar settings using non-PEGylated LPEI polyplexes.[19] Nevertheless, the peak signal (pseudo-colored in red) in positive areas of animals treated with LPEI-PEG polyplexes is between 8×10^3 and 4×10^4 p/sec/cm^2/sr, which is clearly higher than the values obtained with un-shielded LPEI polyplexes in our previous work (4×10^3 p/sec/cm^2/sr). Still, the BLI signal could be correlated with the T2-weighted MRI signal in all cases. Such a correlation could also be observed by Adiseshaiah et al.,[40] where 4T1-luc cells were implanted orthotopically and the lung lesions imaged by BLI and T2-weighted MRI. When compared with PEG polyplexes, transgene activity induced after application of peptide-targeted polyplexes was clearly more pronounced, giving an on average 3-fold higher value (Figures 7 and 8) with peak signals between 8×10^4 and 3×10^5 p/sec/cm^2/sr. Of note, transfection was possible only for tumors accessible via the intratracheal route. In case the tumor obstructed the airways, no transfection of tumor tissue could be achieved (see also Figure S6). In addition, mucus and bronchoalveolar fluid can negatively affect the efficiency of non-viral gene delivery systems in the lung.[44] The excess of positive charge restricts the motility of nanoparticles within a mucous layer[45] or can destabilize particles.[46] Although PEGylation can decrease transfection efficiency *in vitro*, it can be beneficial in overcoming potential barriers *in vivo*: Forier et al.[45] demonstrated that polycation-coated nanoparticles were immobilized when incubated with sputum derived from cystic fibrosis patents, whereas PEGylation significantly improved diffusion of particles. There are rare cases of mucinous breast cancer,[47] although there are no reports that triple-negative breast cancer is producing mucus. Hence delivering nanoparticles via the intratracheal route to tumor nodules accessible from the alveolar site adds another layer of selectivity for gene delivery.[19]

With CD49f targeting, we could observe a strong luciferase signal in the area of the tumor lesion, whereas surrounding, healthy lung tissue appeared not transfected. We conclude that this difference is also due to the fact that also untargeted, unshielded LPEI polyplexes, when applied intratracheally, only very inefficiently transfect healthy lung tissue.[48] For a future therapy approach, a combination therapy of first debulking the tumor mass with standard chemotherapeutics followed

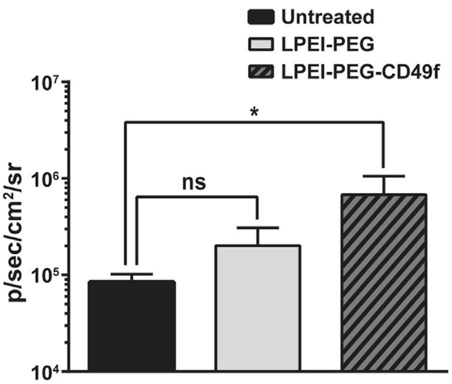

Figure 8. Quantification of BLI Signal after Intratracheal Polyplex Treatment
4T1-iRFP720 tumor-bearing BALB/c mice were treated with indicated polyplexes as described in Figure 7; the BLI signal was quantified in the thoracic area 24 h thereafter and compared with untreated animals. n = 4 + SD. $^*p \leq 0.05$ (Mann-Whitney test).

by CD49f-targeted, anti-CSC-directed therapy seems promising. This could include also delivery of small interfering RNA (siRNA)- or short hairpin RNA (shRNA)-encoding plasmids with selectivity for CSC-related pathways.[49]

Taken together, CD49f targeting of polyplexes with the laminin-derived peptide CYESIKVAVS significantly enhanced transgene expression. Due to its applicability *in vivo* by the intratracheal route, it opens the possibility of efficient transfection of lung tumor lesions also for therapeutic purposes.

MATERIALS AND METHODS

The plasmid pCMV-Gluc was obtained from New England Biolabs (Frankfurt, Germany). The plasmid pCpG-hCMV-EF1α-LucSH encoding for a luciferase-bleomycin fusion protein was already described elsewhere.[50] The *Label*IT Nucleic Acid Labeling Kit, Cy5 was obtained from Mirus Bio (Madison, WI, USA).

MDA-MB-231 (human breast adenocarcinoma; HTB-26), CT26 (murine colon carcinoma; CRL-2638), and 4T1 (murine breast cancer; CRL-2539) cells were obtained from ATCC (LGC Standards, Wesel, Germany). Stably iRFP720-expressing 4T1 cells (4T1-iRFP720) were generated as previously published.[19] MDA-MB-231 were cultured in DMEM high-glucose medium, CT26 in DMEM/F-12 Ham medium, and 4T1-iRFP720 cells in RPMI 1640 medium. All cell culture media were supplemented with 10% fetal bovine serum (FBS; Sigma-Aldrich, Austria), 2% L-glutamine (Sigma-Aldrich; Vienna, Austria), and 1% antibiotics (penicillin/streptomycin; Sigma-Aldrich). Cell culture medium without any supplement is later termed as basal medium.

α-Methyl ω-hydroxy poly(2-ethyl-2-oxazoline) was obtained from Sigma-Aldrich (Austria), and α-OPSS ω-NHS polyethylene glycol (MW 2 kDa) from Rapp Polymere (Tübingen, Germany).

Fmoc protected L-amino acids used for solid-phase peptide synthesis were obtained from Iris Biotech (Germany).

Alexa Fluor 488 phalloidin for actin staining used for CLSM, TrypLE Express for cell detachment, and BCA assay kit for protein quantification were purchased from Thermo Fisher Scientific (Vienna, Austria). Passive lysis buffer was obtained from Promega (Mannheim, Germany).

Native coelenterazine was purchased from Synchem (Felsberg, Germany) and used for evaluating *in vitro* Gluc expression at a concentration of 20 μM in DPBS (supplemented with 5 mM NaCl) as per instructions in Tannous et al.[51] D-luciferin (potassium salt; VivoTrace) was used for *in vivo* BLI at a concentration of 30 mg/mL in DPBS and obtained from Intrace Medical (Lausanne, Switzerland).

For all experiments, water purified with a Sartorius Arium Pro (Göttingen, Germany) system was used.

All other reagents used for synthesis and physical and biological evaluation if not stated differently were obtained from Sigma Aldrich.

All diluents used for nanoparticle analysis by NTA as well as cell culture media were filtered through 0.1-μm cellulose acetate membranes.

Synthesis of LPEI-PEG and LPEI-PEG-CYESIKVAVS (LPEI-PEG-CD49f)

Generation of LPEI-PEG-OPSS was done based on a previously described protocol.[27] In brief, 60 mg of LPEI dissolved in 1.5 mL dry ethanol was mixed with a solution of NHS-PEG-OPSS (2 equiv) in 100 μL DMSO and incubated at 35°C for 3 h under vigorous mixing. The reaction was stopped with 100 μL Tris-HCl buffer (1 M) set to pH 8. After adding 830 μL HEPES/3 M NaCl (pH 7.4), the solution was filled up to a final volume of 5 mL to reach a final NaCl concentration of 0.5 M. Purification of the product was conducted with an Äkta Pure system (GE Healthcare, Vienna, Austria) equipped with a cation exchange resin (MacroPrep High S [Bio-Rad, Vienna, Austria] in HR10/10 column). For removing impurities in the mixture, a mobile phase containing 0.5 M NaCl in HEPES (pH 7.4) was used at a flow rate of 0.5 mL/min for 25 min. Elution of LPEI-PEG-OPSS was done with a NaCl gradient from 0.5 to 3 M in HEPES (pH 7.4) over a total duration of 40 min. LPEI-PEG-OPSS-containing fractions were pooled, dialyzed against water employing a regenerated cellulose membrane, molecular weight cutoff (MWCO): 6–8 kDa (SpectraPor; Repligen, Germany), and lyophilized. The ratio between LPEI and PEG in the product was evaluated by ^{1}H-NMR with a 200-MHz Bruker Avance system (Billerica, MA, USA). Therefore, 5 mg of LPEI-PEG-OPSS was dissolved in 1 mL D_2O and pH was set to 7 with DCl (1 M) and NaOD (1 M). The solvent-derived signal was used as reference (δ = 4.79 ppm) for analysis of signals derived by the conjugate. For determination of PEGylation degree, the peak integral of the signal derived by $\mathbf{CH_2CH_2}$NH was set to 930 (based on a MW of 10 kDa of LPEI). Based on the MW of PEG of 2 kDa, the ratio of LPEI to PEG within the conjugate was calculated. The total amount

of intact OPSS groups in the conjugate was evaluated by adding 15 μL of DTT (1 M in water) to 150 μL of an aqueous LPEI-PEG-OPSS stock with a concentration of 2 mg/mL. The mixture was incubated at room temperature for 10 min and analyzed by UV/Vis spectrophotometry for its absorption at 343 nm generated by 2-thiopyridone released through reductive cleavage of OPSS groups.

For synthesis of LPEI-PEG or LPEI-PEG-CD49f, a 3-fold excess (based on amount of OPSS) of either L-cysteine or CYESIKVAVS dissolved in 30% ACN (in 0.1% trifluoracetic acid) was added to a solution of LPEI-PEG-OPSS in 30% ACN (in 20 mM HEPES, pH 7.4). Unwanted oxidation of the N-terminal thiol group of the peptide was avoided by purging all diluents with argon. The mixture was incubated at room temperature under vigorous stirring. The reaction process was monitored by UV/Vis spectrophotometry and proceeded until no change in the absorption at 343 nm generated by 2-thiopyridone could be detected. A total of 830 μL of 3 M NaCl (in 10% ACN in 20 mM HEPES, pH 7.4) was added to the solution, which was then filled up to a total volume of 5 mL with 10% ACN (in 20 mM HEPES, pH 7.4) to reach a final NaCl concentration of 0.5 M. Purification was again conducted based on cation exchange chromatography using a MacroPrep High S resin (HR10/10 column) with an Äkta Pure system. Uncharged impurities were removed with 0.5 M NaCl (in 10% ACN in 20 mM HEPES, pH 7.4) at a flow rate of 0.5 mL/min for 35 min. LPEI-PEG or LPEI-PEG-CD49f was eluted with a linear NaCl gradient from 0.5 to 3 M (in 10% ACN in 20 mM HEPES, pH 7.4) over 40 min. Conjugate-containing fractions were pooled, dialyzed against water with a regenerated cellulose membrane (MWCO: 6–8 kDa) at 4°C, and lyophilized.

Covalent Labeling of Plasmid DNA with Cy5 Dye

Plasmid pCMV-Gluc was covalently labeled using the *Label*IT Nucleic Acid Labeling Kit, Cy5 (Mirus Bio; Madison, WI, USA) as described previously.[1] In brief, plasmid and reactive dye were incubated in the labeling buffer for 1 h at 37°C. Unbound dye was removed after precipitation of plasmid and subsequent washing steps.

Polyplex Synthesis

Preparation of polyplexes was conducted based on a previously described method.[23] In brief, the same volumes of a pDNA-containing solution and a LPEI/LPEI-PEG/LPEI-PEG-CD49f-containing solution were mixed by flash pipetting. The mixture was then incubated for 5 min at room temperature. For all experiments, polyplexes were prepared at N/P ratio 9 in 20 mM HEPES/5% glucose (HBG; pH 7.4). For *in vitro* experiments, nanoparticles were generated at a final pDNA concentration of 20 μg/mL. For *in vivo* administration, a concentration of 267 μg/mL of formulated pDNA was used.

Evaluation of Nanoparticle Properties (Size and ζ-Potential)

NTA was done based on the instructions by Taschauer et al.[29] Polyplex samples were analyzed for their particle size and ζ-potential on a Malvern NS500 system (UK). For all measurements, dilution factors were chosen to reach 10–100 particles per frame corresponding to a concentration of 10^8–10^9 particles/mL. For size measurement samples were diluted in HBG, and for ζ-potential measurements in 2.5 mM NaCl. Particle size was calculated based on five videos with a duration of 60 s. For analyzing the ζ-potential, a capture duration of 90 s and a secondary duration of 30 s were chosen. All ζ-potential measurements used for evaluation showed a coefficient of correlation of at least 0.95. For analyzing the effect of microspraying with a PennCentury Microsprayer/Syringe Assembly (MSA-250-M; Penn Century, USA) on particle properties, samples were sprayed into centrifuge tubes and analyzed for particle size as described above.

Cell Characterization (CD49f Expression Profile)

Cells (MDA-MB-231/CT26/4T1-iRFP720) were detached with Versene (Thermo Fisher Scientific, Austria) based on the manufacturer's instructions. A total of 2×10^5 cells were transferred into 96-well V-bottom plates and washed with 2 mM EDTA/0.5% (m/v) BSA in DPBS (PEB) to avoid non-specific binding of antibodies. The cell pellet was then resuspended in 20 μL of either PEB (untreated control), a dilution of rat IgG2a κ isotype control (catalog [cat] no. 562602; BD Biosciences, Germany) in PEB, or a dilution of rat anti-human CD49f antibody (cat no. 562582; clone goH3; BD Biosciences; Germany; reactive against human and mouse CD49f) in PEB. Both antibodies used were labeled with BV (Brilliant Violet) 421. Antibody concentrations were chosen as per the manufacturer's instructions. After an incubation period of 30 min at 4°C, cells were washed twice with PEB and resuspended in 100 μL PEB. Flow cytometry was done with a MacsQuant Analyzer 10 system (Miltenyi Biotec, Germany). Live cells were gated and analyzed for their fluorescence signal derived by BV421 in the V1 channel (excitation laser: 405 nm; emission band-pass filter: 450/50 nm).

Cell Association Assay by Flow Cytometry

A total of 5×10^4 MDA-MB-231 cells were seeded into a transparent 96-well plate 24 h prior to treatment. Polyplex samples used for this experimental setup were based on pCMV-Gluc labeled with Cy5. Nanoparticles were prepared as described above. Before sample addition to cells, cell culture medium was exchanged with basal DMEM high-glucose medium. Cells were treated with 1 or 2 μg/mL of formulated pDNA. Both concentrations were tested for a total duration of 30 min and 4 h. Thereafter, cells were washed twice with DPBS, detached with TrypLE Express based on the manufacturer's protocol, resuspended in DPBS, and transferred into a PCR plate (#04-083-0150; Nerbe, Germany). Flow cytometric analysis was conducted with a MacsQuant Analyzer 10 system. For live/dead staining, DAPI was automatically added with an autoinjector reaching a final concentration of 1 μg/mL. Until DAPI addition and automated injection into the flow cytometry system, cell suspensions were permanently kept at 4°C using a cold plate air cooled (CPAC) cooling unit (Inheco, Germany). A total of 1×10^4 live cells were analyzed per well for their Cy5 signal detected in the R1 channel (excitation laser: 635 nm; emission band-pass filter: 655–730 nm). Data evaluation was conducted with FlowJo X software (version: 10.1r5).

Evaluation of Cell Binding and Uptake by CLSM

A total of 5 × 10^4 MDA-MB-231 cells were seeded into chamber slides (Nunc Lab-Tek II 8-well slides; Thermo Fisher Scientific, Germany) 24 h before treatment. Polyplexes were prepared based on Cy5-labeled pCMV-Gluc. Cells were treated with a concentration of 2 μg/mL of formulated pDNA for 4 h in basal DMEM high-glucose medium. After treatment, cells were washed three times with 1% (m/v) BSA (in DPBS) and fixed with 4% (m/v) formaldehyde (in HBS; pH 7.4) for 30 min at room temperature. After three washing steps with 1% (m/v) BSA (in DPBS), cells were permeabilized with 0.1% Triton X-100 (in DPBS) for 5 min and washed again with 1% (m/v) BSA (in DPBS). Actin staining was done with Alexa Fluor 488-phalloidin as per manufacturer's instructions. After three further washing steps with 1% (w/v) BSA (in DPBS), nuclei were stained with DAPI at a concentration of 2 μg/mL for 5 min, and objects were mounted with Vectashield antifade mounting medium (Vector Laboratories, UK). Image acquisition was performed on a Leica TCS SPE microscope (Leica, Germany) with a 63× oil immersion objective. DAPI-derived signal was imaged at 405 nm laser excitation, Alexa Fluor 488-phalloidin at 488 nm, and Cy5 at 635 nm. Detection of emission signal was set for optimal collection of emitted light as per the respective fluorophore. For both magnifications, z scans with a vertical resolution of 0.1 μm were conducted. Data acquisition and analysis were done with LasX software (version 3.1.2.16221).

In Vitro Transfection Assay

For evaluation of transfection efficiency gene delivery, agents (LPEI/LPEI-PEG/LPEI-PEG-CD49f) were tested on MDA-MB-231, CT26, and 4T1-iRFP720. For MDA-MB-231, a seeding density of 2 × 10^4 cells/well was used. Due to their high growth rate, both CT26 and 4T1-iRFP720 were seeded at a density of 1 × 10^4 cells/well. Cell seeding was conducted 24 h before treatment. Polyplexes were based on pCMV-Gluc and tested at pDNA concentrations of 0.5, 1, 2, and 4 μg/mL. Cell treatment was conducted in basal cell culture medium. Four hours after compound addition, supernatant was exchanged with cell culture medium supplemented with 10% FBS, 2% L-glutamine, and antibiotics. After a total treatment time of 24 h, expression of Gluc was analyzed in 20 μL of supernatant. Quantification of light signal was conducted after automated injection of 50 μL of coelenterazine (native; 20 μM in DPBS) with an Infinite M200 Pro system (Tecan Life Sciences, Switzerland) set to an integration time of 10,000 ms. Relative light units (RLUs) were normalized based on total cell count in the case of MDA-MB-231 and CT26. Therefore, cells were washed with DPBS and detached with TrypLE Express based on the manufacturer's protocol. After resuspension of cells in DPBS, cell count was conducted on a MacsQuant Analyzer 10 system. DAPI was used for live/dead staining at a concentration of 1 μg/mL without incubation time. Until automated addition of DAPI followed by injection into the flow cytometer, cell suspensions were permanently kept at 4°C.

Gluc expression signals of 4T1-iRFP720 were normalized to protein amount. Therefore, cells were washed with DPBS and lysed with 30 μL passive lysis buffer (Promega, Germany). Protein quantification was evaluated in 20 μL of cell lysate by BCA kit (Pierce, Thermo Fisher Scientific, Germany) following the manufacturer's instructions.

In Vivo Transfection Assay

In vivo transfection was evaluated on a 4T1-iRFP720 lung tumor model established by Geyer et al.[19] Therefore, female BALB/cJRj (Janvier Labs, France) were kept in individually ventilated Type 2L cages and kept on a low fluorescent diet (AIN-76A, gamma-irradiated; ssniff, Soest, Germany) for at least 10 days before treatment. All procedures were approved by local ethics committee and are in accordance with the Austrian law for the protection of animals and the European Union (EU) directive 2010/63/EU.

4T1-iRFP720 cells were detached with TrypLE Express based on the manufacturer's instructions, washed, and resuspended in DPBS at a final concentration of 10^6 cells/mL. For generating the 4T1-iRFP720 tumor model, 10^5 cells were injected into the lateral tail vein. Tumor growth was monitored by MRI using a small 1.0 Tesla preclinical MRI scanner, equipped with a homogeneous permanent magnet unit (M3 compact MRI System; Aspect Imaging, Shoham, Israel) along with a 50 mm × 38 mm body coil (Mouse Body L50D38 Serial: 1; Aspect Imaging, Shoham, Israel). For all animals, native two-dimensional fast spin echo T2-weighted images were acquired in coronal slice orientation (time to repetition [TR]: 3,250 ms, time to echo [TE]: 63.47 ms, number of slices: 15, slice thickness: 1 mm, number of excitations: 7, slice orientation: coronal, center of slice position: 0°, horizontal field of view [FOV]: 30 mm, vertical FOV: 60 mm, flip angle: 90°, scan time: 5 min 46 s). Mice were anesthetized with 2% isoflurane (Isoflurane CP 1 mL/mL; CP-Pharma, Germany) with an oxygen flow rate of 2 L/min, and eyes were protected by an ointment (Vit A-POS; Ursapharm Arzneimittel, Austria). For optimal acquisition, respiratory movement was detected to suppress motion-derived artifacts. High intrapulmonary tumor load (detected by MRI) was taken as a major criterion for starting the treatment. Image analysis was conducted with VivoQuant software (version 3.0.patch1; Invicro Imaging Service and Software, USA).

Intratracheal administration of polyplex samples was conducted using a Microsprayer/Syringe Assembly (MSA-250-M; Penn Century, USA) as described by Geyer et al.[19,52] Both LPEI-PEG-CD49f and LPEI-PEG were tested as polyplex formulations with pCpG-hCMV-EF1α-LucSH. Polyplexes were prepared at a final pDNA concentration of 267 μg/mL. In total, 20 μg of formulated pDNA was administered into tumor-bearing animals. For microspraying, animals were anesthetized with a mixture of ketamine (80 mg/kg) and xylazine (5 mg/kg). Twenty-four hours after treatment, animals were imaged for bioluminescence signal. Therefore, animals were anesthetized with 2% isoflurane followed by subcutaneous injection of D-luciferin (potassium salt; 30 mg/mL in DPBS; dosage: 120 mg/kg). Bioluminescence signal was collected at an exposure of 3 min in Stage B with an IVIS SpectrumCT system (PerkinElmer, USA) over a duration of 30–45 min. Data were analyzed with Living Image software (version

4.5.2.18424). For evaluation of light signal, a rectangular ROI was placed over the thoracic region.

Histology

Histological evaluation of lungs was conducted based on a previously described protocol.[19,53] In brief, animals were sacrificed directly after BLI and intubated. Instillation fixation was done with 4% formaldehyde (in HBS, pH 7.4). Lungs were subsequently fixed in 4% formaldehyde for 22 h, dehydrated with increasing ethanol concentrations (70%, 96%, 100%), followed by clearing with xylene and embedding in Paraplast. Tissue was rehydrated and sectioned on a microtome (Slee, Germany) with 2-μm thickness. For morphological analysis, tissue was stained for 3 min with Harris-modified hematoxylin (Carl Roth, Germany) followed by treatment with acidified ethanol (1 s), ammonium hydroxide (20 s), and Eosin Y (10 min; Sigma-Aldrich, Austria). For IHC staining, formalin-fixed paraffin-embedded (FFPE) sections were prewarmed for 2 h at 55°C, deparaffinized, and dehydrated, and heat-induced epitope retrieval (HIER) was performed for 30 min in Tris-EDTA buffer (pH 9). After normal serum blockage for 30 min, endogenous avidin and biotin were blocked with Avidin/Biotin Blocking Reagent (BUF016; Bio-Rad) for 15 min each. The samples were incubated with Anti-Integrin α6 antibody [EPR18124] (ab181551; Abcam, UK) (1:100) or Rabbit IgG, monoclonal [EPR25A]–Isotype Control (ab172730; Abcam, UK) in PBS + 2% BSA overnight at 4°C. Endogenous peroxidase was blocked with 0.3% H_2O_2/PBS for 15 min. Samples were further treated via VECTASTAIN ABC HRP Kit (Peroxidase, Rabbit IgG, PK-4001) and counterstained with hematoxylin. The sections were mounted in Entellan (Merck Millipore, Austria) and coverslipped. Images were acquired on an Olympus BX53 light microscope (Olympus, Austria) equipped with an Olympus DP-73 color camera.

Statistical Analysis

Statistical analysis was performed using GraphPad Prism, version 7.02 (GraphPad Software, La Jolla, CA, USA) and an online tool (https://www.socscistatistics.com/).

SUPPLEMENTAL INFORMATION

Supplemental Information can be found online at https://doi.org/10.1016/j.omtn.2019.10.009.

AUTHOR CONTRIBUTIONS

Conceptualization, M.O. and H.S.; Methodology, A.T., W.P. and F.A.; Validation, A.T., H.S. and M.O.; Formal Analysis, A.T., H.S. and M.O.; Investigation, A.T., W.P. F.A., M.B., S.D., T.K., E.G., S.E. and H.S.; Data Curation, M.Z. and E.U.; Writing – Original Draft, A.T., H.S. and M.O.; Writing – Review & Editing, H.S. and M.O.; Visualization, A.T., F.A., M.B., H.S. and M.O.; Supervision, H.S. and M.O.; Project Administration, M.O.; Funding Acquisition, M.O.

ACKNOWLEDGMENTS

We would like to thank Dr. Michal Pechar (Institute of Macromolecular Chemistry, Czech Academy of Science) for help with GPC analysis, Susanne Wiederkum for technical assistance, and Dr. Antonia Geyer for helpful advice concerning intratracheal application. Open access funding was provided by the University of Vienna.

REFERENCES

1. Schäfer, A., Pahnke, A., Schaffert, D., van Weerden, W.M., de Ridder, C.M., Rödl, W., Vetter, A., Spitzweg, C., Kraaij, R., Wagner, E., and Ogris, M. (2011). Disconnecting the yin and yang relation of epidermal growth factor receptor (EGFR)-mediated delivery: a fully synthetic, EGFR-targeted gene transfer system avoiding receptor activation. Hum. Gene Ther. *22*, 1463–1473.
2. Mickler, F.M., Möckl, L., Ruthardt, N., Ogris, M., Wagner, E., and Bräuchle, C. (2012). Tuning nanoparticle uptake: live-cell imaging reveals two distinct endocytosis mechanisms mediated by natural and artificial EGFR targeting ligand. Nano Lett. *12*, 3417–3423.
3. Ge, Z., Chen, Q., Osada, K., Liu, X., Tockary, T.A., Uchida, S., Dirisala, A., Ishii, T., Nomoto, T., Toh, K., et al. (2014). Targeted gene delivery by polyplex micelles with crowded PEG palisade and cRGD moiety for systemic treatment of pancreatic tumors. Biomaterials *35*, 3416–3426.
4. Urnauer, S., Klutz, K., Grünwald, G.K., Morys, S., Schwenk, N., Zach, C., Gildehaus, F.J., Rödl, W., Ogris, M., Wagner, E., and Spitzweg, C. (2017). Systemic tumor-targeted sodium iodide symporter (NIS) gene therapy of hepatocellular carcinoma mediated by B6 peptide polyplexes. J. Gene Med. *19*, e2957.
5. Abourbeh, G., Shir, A., Mishani, E., Ogris, M., Rödl, W., Wagner, E., and Levitzki, A. (2012). PolyIC GE11 polyplex inhibits EGFR-overexpressing tumors. IUBMB Life *64*, 324–330.
6. Boonstra, M.C., de Geus, S.W., Prevoo, H.A., Hawinkels, L.J., van de Velde, C.J., Kuppen, P.J., Vahrmeijer, A.L., and Sier, C.F. (2016). Selecting Targets for Tumor Imaging: An Overview of Cancer-Associated Membrane Proteins. Biomark. Cancer *8*, 119–133.
7. Human Protein Atlas (2019). ITGA6, https://www.proteinatlas.org/ENSG00000091409-ITGA6/tissue.
8. Krebsbach, P.H., and Villa-Diaz, L.G. (2017). The Role of Integrin α6 (CD49f) in Stem Cells: More than a Conserved Biomarker. Stem Cells Dev. *26*, 1090–1099.
9. Yang, Z., Dong, P., Fu, X., Li, Q., Ma, S., Wu, D., Kang, N., Liu, X., Yan, L., and Xiao, R. (2015). CD49f Acts as an Inflammation Sensor to Regulate Differentiation, Adhesion, and Migration of Human Mesenchymal Stem Cells. Stem Cells *33*, 2798–2810.
10. Yu, K.R., Yang, S.R., Jung, J.W., Kim, H., Ko, K., Han, D.W., Park, S.B., Choi, S.W., Kang, S.K., Schöler, H., and Kang, K.S. (2012). CD49f enhances multipotency and maintains stemness through the direct regulation of OCT4 and SOX2. Stem Cells *30*, 876–887.
11. Yamamoto, H., Masters, J.R., Dasgupta, P., Chandra, A., Popert, R., Freeman, A., and Ahmed, A. (2012). CD49f is an efficient marker of monolayer- and spheroid colony-forming cells of the benign and malignant human prostate. PLoS ONE *7*, e46979.
12. Smadja, D.M., Guerin, C.L., Boscolo, E., Bieche, I., Mulliken, J.B., and Bischoff, J. (2014). α6-Integrin is required for the adhesion and vasculogenic potential of hemangioma stem cells. Stem Cells *32*, 684–693.
13. Fukamachi, H., Seol, H.S., Shimada, S., Funasaka, C., Baba, K., Kim, J.H., Park, Y.S., Kim, M.J., Kato, K., Inokuchi, M., et al. (2013). CD49f(high) cells retain sphere-forming and tumor-initiating activities in human gastric tumors. PLoS ONE *8*, e72438.
14. Vassilopoulos, A., Chisholm, C., Lahusen, T., Zheng, H., and Deng, C.X. (2014). A critical role of CD29 and CD49f in mediating metastasis for cancer-initiating cells isolated from a Brca1-associated mouse model of breast cancer. Oncogene *33*, 5477–5482.
15. Ye, F., Qiu, Y., Li, L., Yang, L., Cheng, F., Zhang, H., Wei, B., Zhang, Z., Sun, L., and Bu, H. (2015). The Presence of EpCAM(-)/CD49f(+) Cells in Breast Cancer Is Associated with a Poor Clinical Outcome. J. Breast Cancer *18*, 242–248.
16. Saha, A., Blando, J., Fernandez, I., Kiguchi, K., and DiGiovanni, J. (2016). Linneg Sca-1high CD49fhigh prostate cancer cells derived from the Hi-Myc mouse model are tumor-initiating cells with basal-epithelial characteristics and differentiation potential in vitro and in vivo. Oncotarget *7*, 25194–25207.
17. Wang, Y., Shenouda, S., Baranwal, S., Rathinam, R., Jain, P., Bao, L., Hazari, S., Dash, S., and Alahari, S.K. (2011). Integrin subunits alpha5 and alpha6 regulate cell cycle by

modulating the chk1 and Rb/E2F pathways to affect breast cancer metastasis. Mol. Cancer *10*, 84.

18. DuPré, S.A., Redelman, D., and Hunter, K.W., Jr. (2007). The mouse mammary carcinoma 4T1: characterization of the cellular landscape of primary tumours and metastatic tumour foci. Int. J. Exp. Pathol. *88*, 351–360.

19. Geyer, A., Taschauer, A., Alioglu, F., Anton, M., Maier, J., Drothler, E., Simlinger, M., Yavuz, S., Sami, H., and Ogris, M. (2017). Multimodal Fluorescence and Bioluminescence Imaging Reveals Transfection Potential of Intratracheally Administered Polyplexes for Breast Cancer Lung Metastases. Hum. Gene Ther. *28*, 1202–1213.

20. Tashiro, K., Sephel, G.C., Weeks, B., Sasaki, M., Martin, G.R., Kleinman, H.K., and Yamada, Y. (1989). A synthetic peptide containing the IKVAV sequence from the A chain of laminin mediates cell attachment, migration, and neurite outgrowth. J. Biol. Chem. *264*, 16174–16182.

21. Freitas, V.M., Vilas-Boas, V.F., Pimenta, D.C., Loureiro, V., Juliano, M.A., Carvalho, M.R., Pinheiro, J.J., Camargo, A.C., Moriscot, A.S., Hoffman, M.P., and Jaeger, R.G. (2007). SIKVAV, a laminin alpha1-derived peptide, interacts with integrins and increases protease activity of a human salivary gland adenoid cystic carcinoma cell line through the ERK 1/2 signaling pathway. Am. J. Pathol. *171*, 124–138.

22. Stevenson, M., Hale, A.B., Hale, S.J., Green, N.K., Black, G., Fisher, K.D., Ulbrich, K., Fabra, A., and Seymour, L.W. (2007). Incorporation of a laminin-derived peptide (SIKVAV) on polymer-modified adenovirus permits tumor-specific targeting via alpha6-integrins. Cancer Gene Ther. *14*, 335–345.

23. Rödl, W., Taschauer, A., Schaffert, D., Wagner, E., and Ogris, M. (2019). Synthesis of Polyethylenimine-Based Nanocarriers for Systemic Tumor Targeting of Nucleic Acids. Methods Mol. Biol. *1943*, 83–99.

24. Kikkawa, Y., Hozumi, K., Katagiri, F., Nomizu, M., Kleinman, H.K., and Koblinski, J.E. (2013). Laminin-111-derived peptides and cancer. Cell Adhes. Migr. *7*, 150–159.

25. Li, B., Qiu, T., Zhang, P., Wang, X., Yin, Y., and Li, S. (2014). IKVAV regulates ERK1/2 and Akt signalling pathways in BMMSC population growth and proliferation. Cell Prolif. *47*, 133–145.

26. Chen, X., Fu, W., Cao, X., Jiang, H., Che, X., Xu, X., Ma, B., and Zhang, J. (2018). Peptide SIKVAV-modified chitosan hydrogels promote skin wound healing by accelerating angiogenesis and regulating cytokine secretion. Am. J. Transl. Res. *10*, 4258–4268.

27. Schaffert, D., Kiss, M., Rödl, W., Shir, A., Levitzki, A., Ogris, M., and Wagner, E. (2011). Poly(I:C)-mediated tumor growth suppression in EGF-receptor overexpressing tumors using EGF-polyethylene glycol-linear polyethylenimine as carrier. Pharm. Res. *28*, 731–741.

28. Crosas, E., Egea, M.A., and Reig, F. (2006). Spectroscopic techniques applied to the study of laminin fragments inserted into model membranes. J. Colloid Interface Sci. *295*, 264–269.

29. Taschauer, A., Geyer, A., Gehrig, S., Maier, J., Sami, H., and Ogris, M. (2016). Up-Scaled Synthesis and Characterization of Nonviral Gene Delivery Particles for Transient In Vitro and In Vivo Transgene Expression. Hum. Gene Ther. Methods *27*, 87–97.

30. Klutz, K., Schaffert, D., Willhauck, M.J., Grünwald, G.K., Haase, R., Wunderlich, N., Zach, C., Gildehaus, F.J., Senekowitsch-Schmidtke, R., Göke, B., et al. (2011). Epidermal growth factor receptor-targeted (131)I-therapy of liver cancer following systemic delivery of the sodium iodide symporter gene. Mol. Ther. *19*, 676–685.

31. Brooks, D.L., Schwab, L.P., Krutilina, R., Parke, D.N., Sethuraman, A., Hoogewijs, D., Schörg, A., Gotwald, L., Fan, M., Wenger, R.H., and Seagroves, T.N. (2016). ITGA6 is directly regulated by hypoxia-inducible factors and enriches for cancer stem cell activity and invasion in metastatic breast cancer models. Mol. Cancer *15*, 26.

32. Hu, T., Zhou, R., Zhao, Y., and Wu, G. (2016). Integrin α6/Akt/Erk signaling is essential for human breast cancer resistance to radiotherapy. Sci. Rep. *6*, 33376.

33. von Gersdorff, K., Sanders, N.N., Vandenbroucke, R., De Smedt, S.C., Wagner, E., and Ogris, M. (2006). The internalization route resulting in successful gene expression depends on both cell line and polyethylenimine polyplex type. Mol. Ther. *14*, 745–753.

34. Wightman, L., Kircheis, R., Rössler, V., Carotta, S., Ruzicka, R., Kursa, M., and Wagner, E. (2001). Different behavior of branched and linear polyethylenimine for gene delivery in vitro and in vivo. J. Gene Med. *3*, 362–372.

35. de Bruin, K., Ruthardt, N., von Gersdorff, K., Bausinger, R., Wagner, E., Ogris, M., and Bräuchle, C. (2007). Cellular dynamics of EGF receptor-targeted synthetic viruses. Mol. Ther. *15*, 1297–1305.

36. Degeling, M.H., Bovenberg, M.S., Lewandrowski, G.K., de Gooijer, M.C., Vleggeert-Lankamp, C.L., Tannous, M., Maguire, C.A., and Tannous, B.A. (2013). Directed molecular evolution reveals Gaussia luciferase variants with enhanced light output stability. Anal. Chem. *85*, 3006–3012.

37. Shorter, S.A., Gollings, A.S., Gorringe-Pattrick, M.A.M., Coakley, J.E., Dyer, P.D.R., and Richardson, S.C.W. (2017). The potential of toxin-based drug delivery systems for enhanced nucleic acid therapeutic delivery. Expert Opin. Drug Deliv. *14*, 685–696.

38. Jin, L., Han, B., Siegel, E., Cui, Y., Giuliano, A., and Cui, X. (2018). Breast cancer lung metastasis: Molecular biology and therapeutic implications. Cancer Biol. Ther. *19*, 858–868.

39. Rashid, O.M., Nagahashi, M., Ramachandran, S., Dumur, C.I., Schaum, J.C., Yamada, A., Aoyagi, T., Milstien, S., Spiegel, S., and Takabe, K. (2013). Is tail vein injection a relevant breast cancer lung metastasis model? J. Thorac. Dis. *5*, 385–392.

40. Adiseshaiah, P.P., Patel, N.L., Ileva, L.V., Kalen, J.D., Haines, D.C., and McNeil, S.E. (2014). Longitudinal imaging of cancer cell metastases in two preclinical models: a correlation of noninvasive imaging to histopathology. Int. J. Mol. Imaging *2014*, 102702.

41. Storti, C., Le Noci, V., Sommariva, M., Tagliabue, E., Balsari, A., and Sfondrini, L. (2015). Aerosol Delivery in the Treatment of Lung Cancer. Curr. Cancer Drug Targets *15*, 604–612.

42. Jia, S.F., Worth, L.L., Densmore, C.L., Xu, B., Duan, X., and Kleinerman, E.S. (2003). Aerosol gene therapy with PEI: IL-12 eradicates osteosarcoma lung metastases. Clin. Cancer Res. *9*, 3462–3468.

43. Hasenpusch, G., Pfeifer, C., Aneja, M.K., Wagner, K., Reinhardt, D., Gilon, M., Ohana, P., Hochberg, A., and Rudolph, C. (2011). Aerosolized BC-819 inhibits primary but not secondary lung cancer growth. PLoS ONE *6*, e20760.

44. Sanders, N., Rudolph, C., Braeckmans, K., De Smedt, S.C., and Demeester, J. (2009). Extracellular barriers in respiratory gene therapy. Adv. Drug Deliv. Rev. *61*, 115–127.

45. Forier, K., Messiaen, A.S., Raemdonck, K., Deschout, H., Rejman, J., De Baets, F., Nelis, H., De Smedt, S.C., Demeester, J., Coenye, T., and Braeckmans, K. (2013). Transport of nanoparticles in cystic fibrosis sputum and bacterial biofilms by single-particle tracking microscopy. Nanomedicine (Lond.) *8*, 935–949.

46. Thomsen, T.B., Li, L., and Howard, K.A. (2014). Mucus barrier-triggered disassembly of siRNA nanocarriers. Nanoscale *6*, 12547–12554.

47. Hanagiri, T., Ono, K., Baba, T., So, T., Yamasaki, M., Nagata, Y., Uramoto, H., Takenoyama, M., and Yasumoto, K. (2010). Clinicopathologic characteristics of mucinous carcinoma of the breast. Int. Surg. *95*, 126–129.

48. Bragonzi, A., Dina, G., Villa, A., Calori, G., Biffi, A., Bordignon, C., Assael, B.M., and Conese, M. (2000). Biodistribution and transgene expression with nonviral cationic vector/DNA complexes in the lungs. Gene Ther. *7*, 1753–1760.

49. Srinivasan, M., Bharali, D.J., Sudha, T., Khedr, M., Guest, I., Sell, S., Glinsky, G.V., and Mousa, S.A. (2017). Downregulation of Bmi1 in breast cancer stem cells suppresses tumor growth and proliferation. Oncotarget *8*, 38731–38742.

50. Magnusson, T., Haase, R., Schleef, M., Wagner, E., and Ogris, M. (2011). Sustained, high transgene expression in liver with plasmid vectors using optimized promoter-enhancer combinations. J. Gene Med. *13*, 382–391.

51. Tannous, B.A. (2009). Gaussia luciferase reporter assay for monitoring biological processes in culture and in vivo. Nat. Protoc. *4*, 582–591.

52. Geyer, A., Lorenzer, C., Gehrig, S., Simlinger, M., Winkler, J., Sami, H., and Ogris, M. (2017). Fluorescence- and computed tomography for assessing the biodistribution of siRNA after intratracheal application in mice. Int. J. Pharm. *525*, 359–366.

53. Braber, S., Verheijden, K.A.T., Henricks, P.A.J., Kraneveld, A.D., and Folkerts, G. (2010). A comparison of fixation methods on lung morphology in a murine model of emphysema. Am. J. Physiol. Lung Cell. Mol. Physiol. *299*, L843–L851.

OMTN, Volume *18*

Supplemental Information

Peptide-Targeted Polyplexes for Aerosol-Mediated Gene Delivery to CD49f-Overexpressing Tumor Lesions in Lung

Alexander Taschauer, Wolfram Polzer, Fatih Alioglu, Magdalena Billerhart, Simon Decker, Theresa Kittelmann, Emanuela Geppl, Salma Elmenofi, Martin Zehl, Ernst Urban, Haider Sami, and Manfred Ogris

Supplemental Figures

Supp. Figure 1

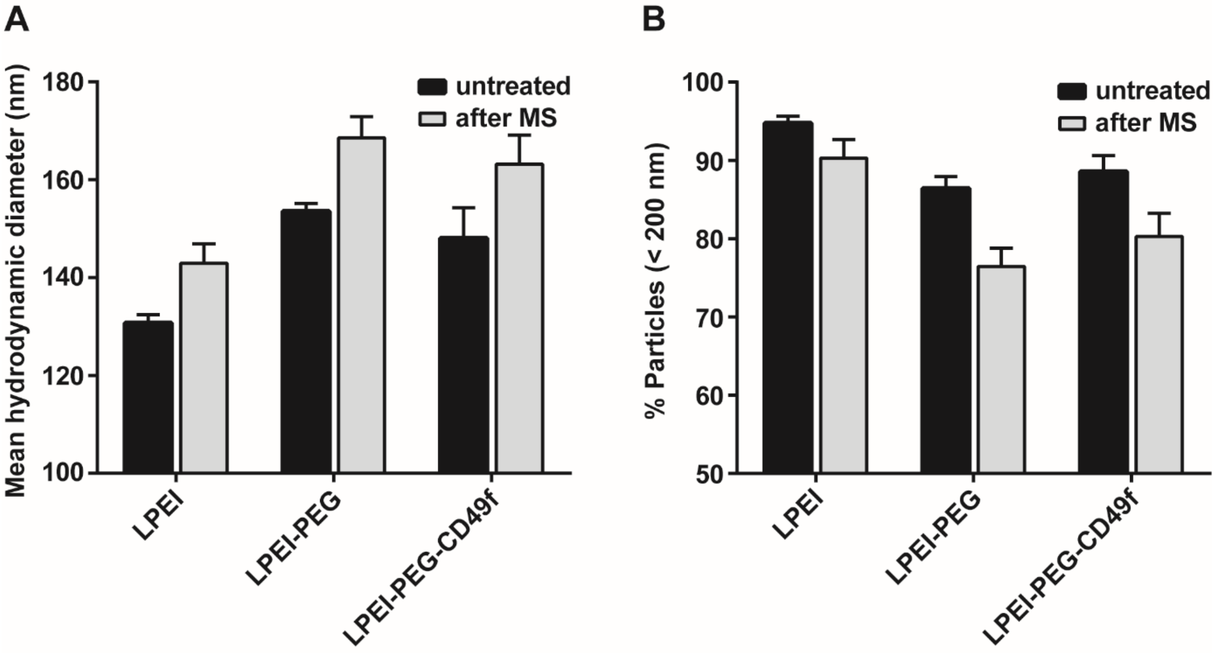

Supp. Figure 1: NTA analysis of polyplexes after aerosolization. Both mean hydrodynamic diameter (A) and percentage of particles <200 nm (B) were evaluated with polyplexes based on LPEI, LPEI-PEG and LPEI-PEG-CD49f at N/P 9 before and after aerosolization. Values are depicted as mean values (n=3 + stddev).

Supp. Figure 2

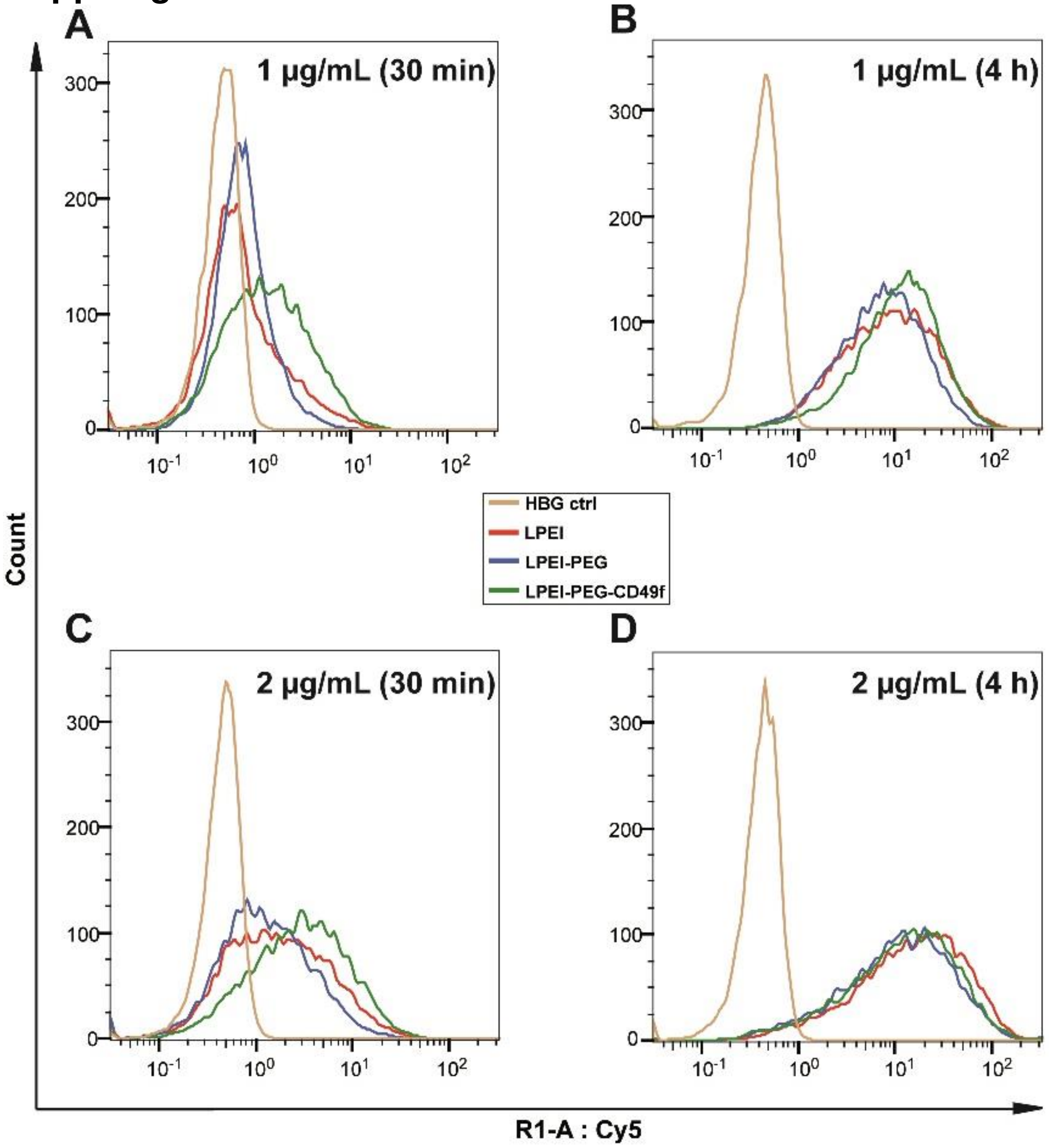

Supp. Figure 2: Cell association of polyplexes. MDA-MB-231 cells were incubated with polyplexes prepared at 20 µg/mL and N/P 9 with Cy5-labelled pDNA diluted in basal medium to a final concentration of 1 µg/mL (A, B) or 2 µg/mL (C,D) for 30 min (A,C) or 4 h (B,D). After incubation, cells were harvested and analyzed by flow cytometry. Data is depicted as representative histograms showing the Cy5 derived fluorescence signal of live cells in the R1 channel (Cy5 fluorescence).

Supp. Figure 3

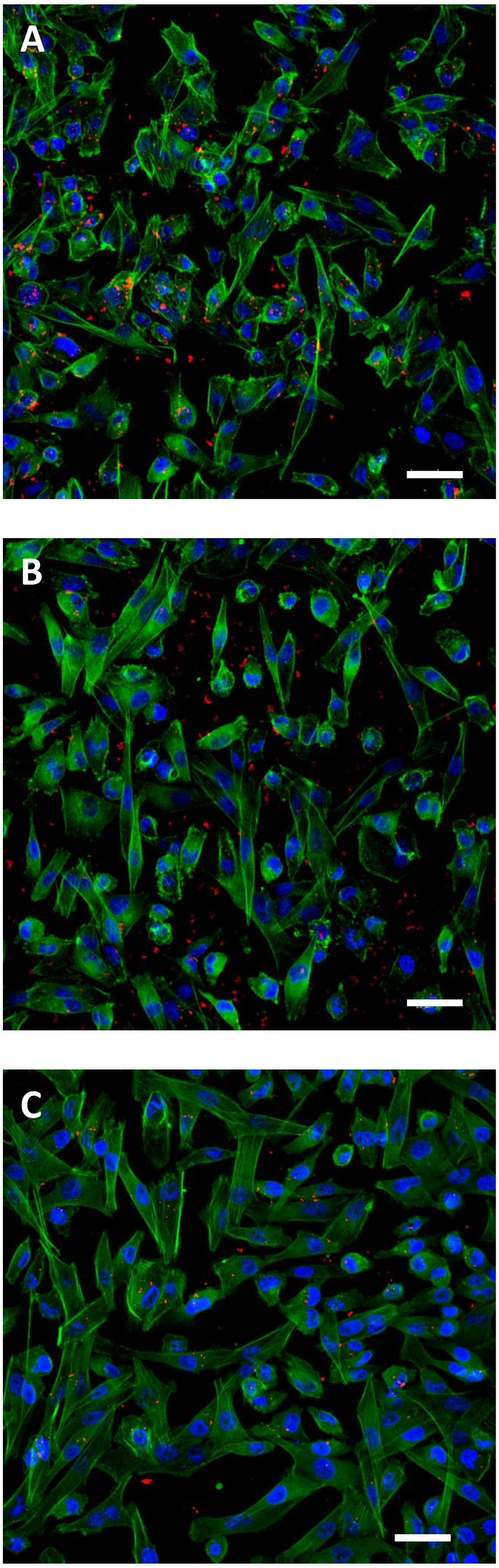

Supp. Figure 3: Cellular association of polyplexes studied by CLSM. MDA-MB-231 cells were incubated in basal cell culture medium with Cy5-labelled polyplexes (red) prepared at N/P 9 at a pDNA concentration of 2 µg/mL for 4 h. Thereafter cells were fixed, stained with AlexaFluor® 488-phalloidin (green) and DAPI (blue). (A) LPEI, (B) LPEI-PEG and (C) LPEI-PEG-CD49f polyplexes. CLSM imaging was conducted with a 20X oil objective; maximum intensity projections of z-sections are shown. Scale bars: 40 µm.

Supp. Figure 4

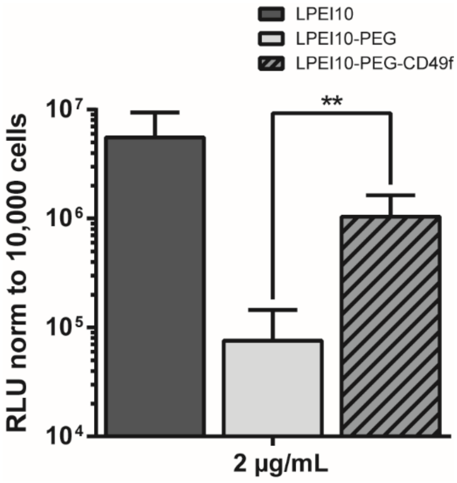

Supp. Figure 4: Transfection study with CT26 cells. CT26 were seeded in 96-well plates and treated with polyplexes based on pCMV-Gluc at 2 µg/mL. 4 h after polyplex addition cellular supernatant was exchanged with cell culture medium supplemented with FCS, L-glutamine and antibiotics. 24 h after starting cell treatment Gaussia luciferase was quantified in the supernatant and RLU values were normalized based on total count of live cells. Data is depicted as mean values (n=3 + stddev; data from 2 independent experiments; ** $p \leq 0.01$ (Mann-Whitney))

Supp. Figure 5

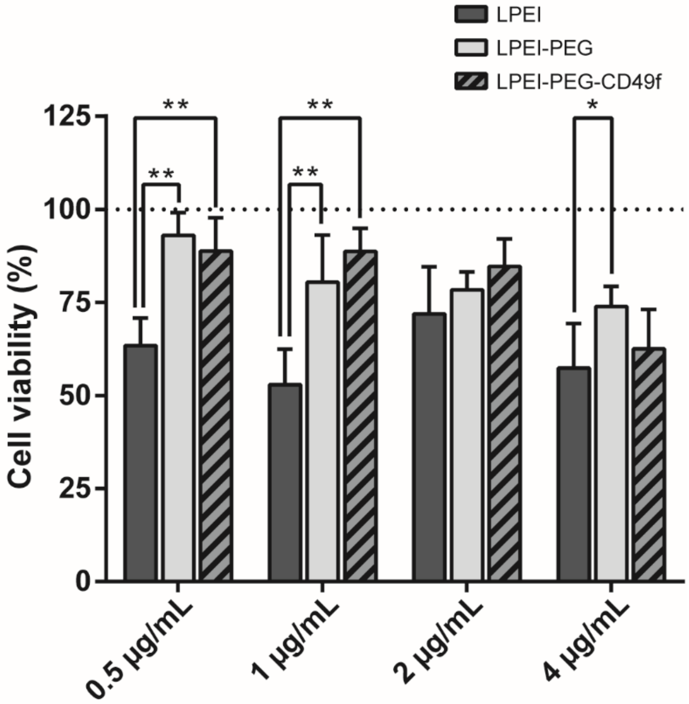

Supp. Figure 5: Cell viability studies by flow cytometry. MDA-MB-231 cells were treated with polyplexes at 4 different concentrations (0.5 µg/mL; 1 µg/mL; 2 µg/mL; 4 µg/mL). 24 h post treatment live cells were counted by flow cytometry after DAPI addition. DAPI derived fluorescence signal was detected in the V1 channel. Total count of live cells was normalized based on buffer treated cells. Data is depicted as mean values (n=3 + stddev; data from 2 independent experiments; *$p \leq 0.05$, ** $p \leq 0.01$ (Mann-Whitney))

Supp. Figure 6

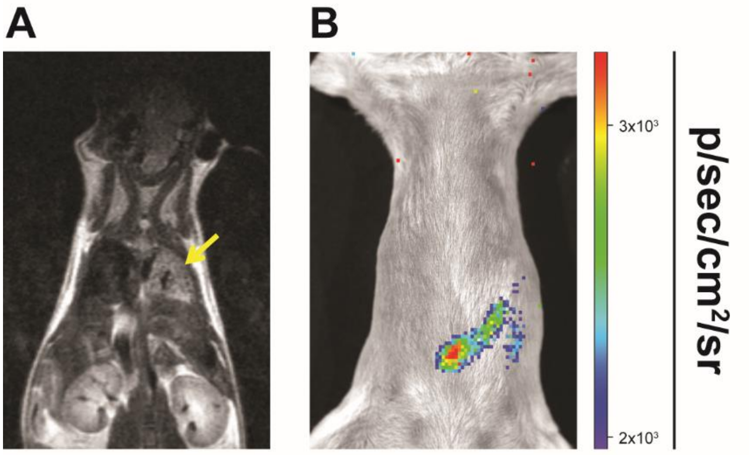

Supp. Figure 6: Representative example of intratracheal polyplex treatment of 4T1-iRFP720 tumor bearing animal with inaccessible tumor tissue. MRI shows intense tumor growth in the whole left lung (marked with yellow arrow) (A). Treatment with LPEI-PEG-CD49f based polyplexes of this animal did not result in sufficient transfection (B). Background BLI signal (color coded) in the abdominal region is depicted as overlay on a reflected light picture.

Supplementary videos:

Video S1:
MRI (T2) coronal fly-through, tumor free control animal shown in Figure 6A

Video S2:
MRI (T2) coronal fly-through, 4T1 tumor bearing animal shown in Figure 6B and Figure 7G

Video S3:
MRI (T2) coronal fly-through, 4T1 tumor bearing animal shown in Figure 7A

Video S4:
MRI (T2) coronal fly-through, 4T1 tumor bearing animal shown in Figure 7C

Video S5:
MRI (T2) coronal fly-through, 4T1 tumor bearing animal shown in Figure 7E

Video S6:
MRI (T2) coronal fly-through, 4T1 tumor bearing animal shown in Figure 7I

Video S7
MRI (T2) coronal fly-through, 4T1 tumor bearing animal shown in Figure 7K

Video S8:
MRI (T2) coronal fly-through, 4T1 tumor bearing animal shown in Supp. Figure 6A

Supplemental methods

Synthesis of linear polyethylenimine (LPEI; Mw 10 kDa)

α-Methyl ω-hydroxy LPEI (Mw 10 kDa) was generated based on a previously described protocol.[1] 2 g α-Methyl ω-hydroxy poly(2-ethyl-2-oxazoline) was dissolved in 50 mL HCl (7 M) and heated overnight under reflux. LPEI precipitated as HCl salt and was purified by centrifugation with HCl (7 M). After dissolving the precipitate in 30 mL water the solution was again heated under reflux and pH was adjusted to 12 with NaOH (1 M). The mixture was cooled to room temperature where LPEI formed a white precipitate as free base. The precipitate was then washed by centrifugation with NaOH (1 M) and water. After resuspending in water the product was lyophilized. Analysis of both structure and purity of LPEI was conducted by ^{1}H-NMR on a Bruker Avance (200 MHz; US) system. Therefore, 5 mg of the product were dissolved in $CDCl_3$. The peak derived by the solvent was used as reference (δ [chemical shift]=7.24 ppm) for evaluation of signals derived from C**H**$_2$-C**H**$_2$-NH and CH_2-CH_2-N**H**. α-Methyl ω-hydroxy poly(2-ethyl-2-oxazoline) was used as control compound for showing complete hydrolysis. GPC analysis was conducted with a GPC/HPLC system equipped with a Catsec-300 column, a UV-Vis and a multiangle light scattering DAWN EOS (Wyatt; Germany) detector. GPC was done with 0.1 % TFA at a flow rate of 0.2 mL/min. For preparing stock solutions used for conjugate synthesis or polyplex generation LPEI was resuspended in water and pH was set to 7.4 with HCl and NaOH. The resulting product was then filtered through a 0.2 µm cellulose acetate membrane. Concentration of LPEI content in aqueous solutions was determined by $CuSO_4$ assay based on UV/Vis spectrophotometry like described elsewhere.[2] For long term storage LPEI was either stored as lyophilized powder under dry conditions at room temperature or dissolved in water at pH 7.4 at -80 °C.

Peptide synthesis

The peptide with the amino acid sequence CYESIKVAVS was synthesized on a semi-automated Biotage Initiator+ system (Sweden). The synthesis setup was based on using Fmoc protected L-amino acids and a ChemMatrix® resin functionalized with Rink-amide linker as solid phase. For every coupling step amino acids were C-terminally activated with 1-hydroxybenzotriazole hydrate (HOBt; 1 eq based on amino acid) and 2-(1H-Benzotriazole-1-yl)-1,1,3,3-tetramethylaminium tetrafluoroborate (TBTU; 1 eq based on amino acid) under addition of N,N-Diisopropylethylamine (DIPEA; 2 eq based on amino acid). The C-terminal amino acid (Fmoc-L-Serin-tBu) was used in a five-fold excess based on the binding capacity of the resin. All following activated amino acids were used in a 2.5-fold excess. Coupling steps of all activated amino acids except activated Fmoc-L-cysteine-Trt were conducted at a temperature of 75 °C for 10 minutes. Coupling of activated Fmoc-L-cysteine-Trt was done twice at 45 °C for 10 minutes. For detection of free terminal primary amino groups Kaiser (ninhydrin) test was conducted after each coupling step.[3] Deprotection of the N-terminus of the growing peptide chain was conducted with piperidine (20 % (V/V) in DMF) twice for 3 minutes at a temperature of 75 °C. Cleavage of the peptide from the resin was done with trifluoracetic acid (TFA):phenol:water:triisopropylsilane (88:5:5:2) for 4 hours at room temperature under vigorous mixing. The solution was then concentrated under vacuum and the product was precipitated with cold diethyl ether. After washing the precipitate with diethyl ether by centrifugation the product was redissolved in water and lyophilized. Purification was conducted by reversed phase chromatography on a Shimadzu HPLC system (Austria) equipped with a Zorbax® 5B-C18 column (21.2 mm x 25 cm). For purification a linear gradient with an increasing concentration of acetonitrile (acidified with 0.1 % TFA) in water (acidified with 0.1 % TFA) starting from 5 to 50 % over 30 minutes at a flow rate of 21 mL/min was chosen. Detection was done at a wavelength of 215 nm. Fractions containing CYESIKVAVS were pooled and lyophilized. Peptide purity was analyzed both by HPLC and high resolution mass spectrometry (HRMS). The analytical HPLC system was equipped with a Thermoquest hypersil division C18 column (Thermo Fisher scientific; Germany) and the same gradient as described before at a flow rate of 1 mL/min was used. HRMS was employed for analysis of purity and amino acid sequence at a sample concentration of 10 µg/mL in 50 % (V/V) ACN (in 0.1 % (V/V) formic acid) with a Bruker maXis Hd system (US). Peptide sequencing was conducted based on single and double charged b- and y-fragments. For further procedure only CYESIKVAVS with a purity of >95 % (based on HPLC) was used.

Supplemental References

1. Rödl, W, Schaffert, D, Wagner, E, and Ogris, M (2013). Synthesis of polyethylenimine-based nanocarriers for systemic tumor targeting of nucleic acids. *Methods Mol Biol* **948**: 105-120.

2. Ungaro, F, De Rosa, G, Miro, A, and Quaglia, F (2003). Spectrophotometric determination of polyethylenimine in the presence of an oligonucleotide for the characterization of controlled release formulations. *J Pharm Biomed Anal* **31**: 143-149.

3. Kaiser, E, Colescott, RL, Bossinger, CD, and Cook, PI (1970). Color test for detection of free terminal amino groups in the solid-phase synthesis of peptides. *Analytical Biochemistry* **34**: 595-598.
